# Evaluation of Public–Private Partnership in the Veterinary Domain Using Impact Pathway Methodology: In-depth Case Study in the Poultry Sector in Ethiopia

**DOI:** 10.3389/fvets.2022.735269

**Published:** 2022-02-22

**Authors:** N'gbocho Bernard N'Guessan, Mariline Poupaud, Isabelle Dieuzy-Labaye, Yohannes T. Asfaw, Barbara Wieland, Fseha Tesfu, Ulric Daniel, Phitsanu Tulayakul, Marisa Peyre

**Affiliations:** ^1^Faculty of Veterinary Medicine, Kasetsart University, Bangkok, Thailand; ^2^UMR ASTRE, Univ Montpellier, CIRAD, INRAE, Montpellier, France; ^3^Fundamental and Applied Research for Animals and Health (FARAH), University of Liège, Liège, Belgium; ^4^World Organisation for Animal Health (OIE), Paris, France; ^5^International Livestock Research Institute, Addis Ababa, Ethiopia; ^6^Institute of Virology and Immunology, Mittelhaeusern, Switzerland; ^7^EthioChicken, Addis Ababa, Ethiopia

**Keywords:** evaluation, impact pathway, public-private-partnership, participatory approaches, poultry, Ethiopia

## Abstract

Public–private partnerships (PPPs) in the veterinary domain are joint approaches in which public veterinary services and private actors such as private veterinarians, producers' associations, or private companies work together to address complex animal health challenges. They are implemented worldwide and can help to strengthen the capacities of veterinary services, but few have been evaluated. None of the evaluations developed in the veterinary domain explicitly addressed PPPs, their complex program design, their evolving governance, and coordination system, and their impacts. This work represents the first application of the participatory impact pathway methodology for the evaluation of a PPP in the veterinary domain. The PPP evaluated aimed at developing the poultry sector in Ethiopia and improving poultry health service coverage, particularly in remote areas. The combination of semi-structured interviews (*n* = 64) and collective reflection during three workshops (*n* participants = 26, 48, 18), captured the viewpoints of public and private partners, actors who influenced the partnership, and actors impacted by it. The context of the PPP was analyzed, and the causal relationships between the PPP and its impacts were investigated. This work showed that collaboration between the public and private sector occurred at several administrative levels. The actors considered a variety of impacts, on the economy, business, trust, and health, which were then measured through different indicators. The actors also identified the added value of the PPP to enrich those impacts. The participatory impact pathway methodology helped to strengthen the engagement of actors in the PPP and to formulate recommendations at the policy level to favor positive results. This case study represents a milestone in building a participatory evaluation framework of PPP in the veterinary domain.

## Introduction

Public–private partnership (PPP) in the veterinary domain^1^ is defined by the World Organization for Animal Health (OIE) as “a joint approach in which the public and private sectors agree responsibilities and share resources and risks to achieve common objectives that deliver benefits in a sustainable manner” (1). Through PPPs, the public Veterinary Services and private actors, such as private veterinarians, producers' associations, or private companies, work together to address complex animal health challenges. PPPs may represent a means of strengthening the veterinary services^2^ and improving animal health programs (2). The establishment of effective PPPs can contribute to a more efficient use of available resources or an extension of veterinary health coverage, particularly in remote areas (1, 3). The examples of the risks of PPPs include conflict of interests, administrative burden, or a lack of funding availability (4). Galière et al. (4), analyzed 97 PPPs implemented across the world, described in detail through an online questionnaire. Three PPP clusters were identified. These clusters are largely conditioned by the type of private actor (4). Cluster 1, “transactional PPPs”, are often initiated and financed by the public sector, and the services come from private veterinarians or paraprofessionals who are contracted or given a sanitary mandate. Cluster 2, “collaborative PPPs”, correspond to PPPs usually motivated by trade, exports, and/or commercial interests. These PPPs are initiated by both the private sector, often represented by producer associations, and the public sector. Finally, Cluster 3, “transformative PPPs”, corresponds to PPPs focused on establishing the capability to deliver otherwise unattainable major programs. They are initiated and financed by the private sector (local or international companies) but sanctioned by, and working with, the national Veterinary Services (1).

One of the PPPs described in the article by Galière et al. (4), belonging to the “transformative” cluster, is implemented in Ethiopia since 2010, with the aim of developing the poultry sector. This PPP represents a collaboration between a company raising day-old chicks and producing feed, EthioChicken, and the public Veterinary Services of Ethiopia. EthioChicken raises poultry parental stock and produces genetically improved day-old chicks (hybrid breed for meat and egg production) in Ethiopia. The day-old chicks are then raised to 45 days old by agents. The grower agents are trained by EthioChicken, and they provide the chicks with poultry healthcare, such as vaccination. These 45 day-old chickens are delivered to smallholder farmers *via* a distribution network developed through PPPs between EthioChicken and the national and regional public Veterinary Services, under the supervision of the Ministry of Livestock and Fisheries.^3^ The public Veterinary Services also provide poultry health services at the local level (4).

In Ethiopia, more than 22 million people, representing 20% of the total population, live below the national poverty line (5). The Ethiopian economy is primarily based on agriculture, which provides 85% employment and contributes to around 45% of gross domestic product and 62% of total exports (5). In 2018, the total poultry population was estimated to be about 57 million (6). Rural poultry production is mainly based on the traditional family poultry system with indigenous breeds, which represents 78.8% of the total poultry population (6). The average consumption of poultry meat is relatively low (600 g/person/annum) compared to other African countries (average of 2 kg/person/annum), which is partly due to a low poultry production in the country. Since 2006, there has been a growing demand for chicken meat in urban areas in Ethiopia due to the increase of beef and sheep meat prices (7). The Ethiopian government plays a role in the development of agriculture in order to reduce the poverty and malnutrition rate. Since 2015, the Ethiopian government, through the Ethiopian Livestock Master Plan 2015–2020, aims at increasing Ethiopians' production and consumption of poultry meat and eggs by developing improved family poultry production systems and specialized layer and broiler production systems (8). As an example, the exotic breed in Ethiopia produces 128 eggs of the eggs per hen and per period, while the hybrid breed produces 48 and the indigenous 13 (6). The government planned to meet these targets “by providing incentives to the private sector for poultry investment, strengthening research to select productive indigenous breeds, and by developing breeds suitable for improved family poultry production systems” (8). The PPP between EthioChicken and the public Veterinary Services aimed to help increase poultry production in Ethiopia by providing 45-day-old chicken and poultry health support to smallholder farmers.

Despite many examples of PPPs implemented in the veterinary domain, few studies have evaluated the initiatives in place. Evaluation is an important step for any programs: it helps in planning, redefining strategies, taking appropriate corrective actions, and optimizing resources (9). Evaluation is also a means of reinforcing partnerships and the process of collaboration and ensuring trust between partners (10). Most evaluations mobilized in the veterinary domain are technical or efficiency evaluations, characterized, for example, by avoided losses in animal production (11). Some evaluations, particularly those applied to surveillance programs, have also focused on the process (or functioning) of the programs by examining the conditions under which the program operates and the organizational elements (12, 13). However, none of the evaluation in the veterinary domain explicitly addressed PPPs and their impacts. In the case of PPPs, involving multi-actor collaboration, complex program design, an evolving governance and coordination system, uncertain program evolution, and a diversity of possible impacts, the evaluations mobilized to date in the veterinary domain do not appear to be fully adequate. impact pathway methodology has been developed in agricultural development evaluation. The idea was is to complement existing economic impact assessment methods and to gain insight into the non-linear mechanisms leading to impacts. This methodology analyses how programs are built and attempts to make explicit the complex causal relationship between the programs and the impacts. The methodology also assesses and measures impacts, normally several years after the program has finished, as the impacts are what remain after the program's ending (14). To our knowledge, this methodology had neither been previously used to evaluate PPPs in the veterinary domain, nor to evaluate other programs in the veterinary domain.

The general objective of this study is to discuss the interest and challenges of the participatory impact pathway methodology for evaluating a PPP in the veterinary domain. To do so, we applied this methodology to evaluate the PPP between EthioChicken and the public Veterinary Services of Ethiopia. Seeking to understand the contribution of PPPs to impacts, the mapping of actors was described, the causal relationships between the inputs of the PPP and the impacts clarified, and then the impacts measured.

## Materials and Methods

### The Participatory Impact Pathway

In order to evaluate a PPP in the veterinary domain, we adapted the participatory impact pathway methodology “ImpresS”, developed to evaluate research projects by the French Agricultural Research Center for International Development (CIRAD*)* (15), itself inspired by pre-existing methodologies (14, 16, 17). As the PPP evaluated is still active, we used the guidelines for *in itinere* evaluation (ex-post evaluation takes place when the program is completed). ImpresS methodology is a participatory evaluation methods (18). Participatory evaluation considers a plurality of viewpoints, thereby improving the understanding a complex, multi-stakeholder program such as the PPP. The participatory evaluation also promotes the formulation of locally relevant evaluation questions, supports for collective learning, and enhances the acceptability of evaluation recommendations by targeted stakeholders (19–21).

#### The Definition of Impact Pathway

**Figure 1:**
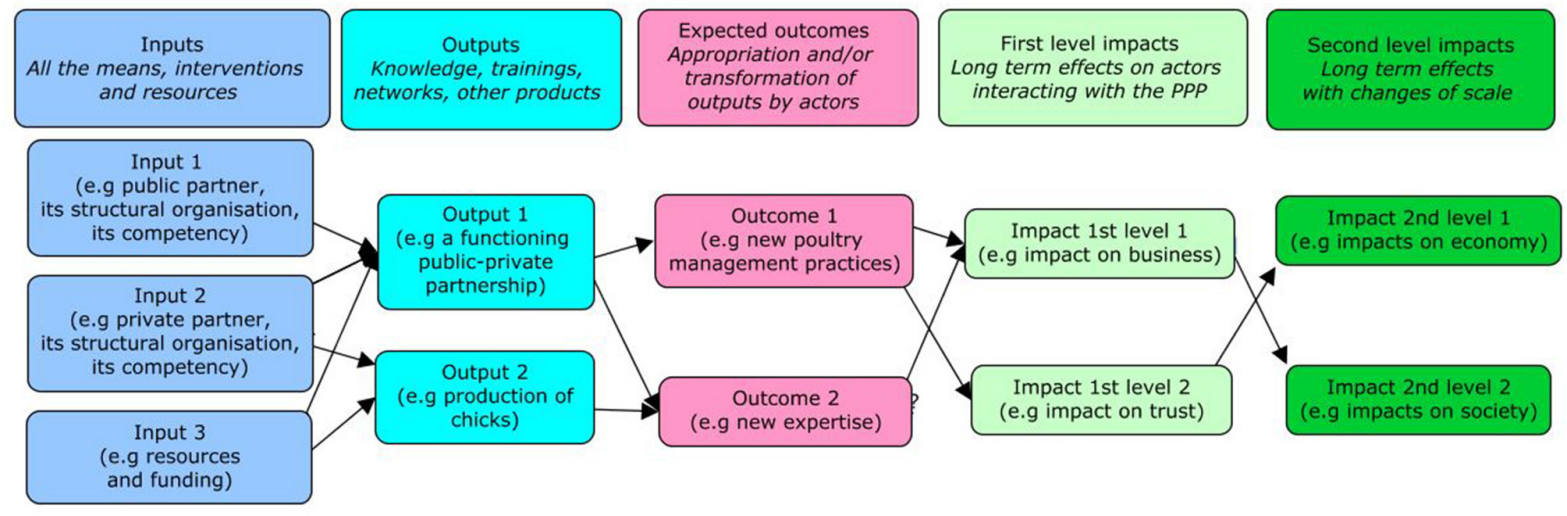
Simplified graphic of an impact pathway. Some hypothesis were made on the potential inputs, outputs, outcomes, and impacts of the PPP evaluated to illustrate the impact pathway.

An impact pathway is based on a program theory, which is an explicit model of how a program will, or has, brought about impacts. The impact pathway makes it possible to determine the complex cause-and-effect relationships between a programme such as PPP and its impacts. The main objective of developing the impact pathway is to demonstrate the extent to which a programme contributes to impacts by looking at the change that it brings for actors and then the economic, social, environmental, and other impacts that these changes produce. The impact pathway distinguishes between outputs (activity or products that result directly from the programme) and outcomes, which correspond to the appropriation and/or transformation of the outputs by the actors, these outcomes being translated into impacts (see Box 1 for a more precise definition) (Figure 1).

Box 1 **Definition of inputs, outputs, outcomes, and impacts.**

*Inputs* encompass all the means (interventions and resources) that make it possible to undertake a program (human and material resources, budget, information, tacit or pre-existing knowledge, other activities, etc.) and thus generate results (outputs).

*Outputs* can take the form of knowledge, professional or academic training, expertise, technology, network, or other forms of products.

*Outcomes* correspond to an appropriation and/or transformation of program outputs by stakeholders, leading to new practices (agricultural or managerial), new organizations, or new rules (15).

*Impacts* are the long-term effects induced by a program. They are what remains after the program is completed. The impacts could be of multiple natures (e.g., economic, social, sanitary, political), at various levels (e.g., individual, institutional, regional, national, global) and of different types (positive or negative; direct or indirect) (15).

For PPPs in the veterinary domain, they can be of different types: economic, societal, related to business, health, or trust and can be measured by indicators (2).

The impacts can be characterized by intensity and magnitude through indicators. Intensity reflects the degree of change attributed to the program and observed for a given impact, while magnitude reflects the extent or spread of the change (e.g., the number of producers affected by the change).First-level impacts are measured on actors interacting directly or indirectly with the program and can be evaluated with these actors. Second-level impacts result from the changes of scale (e.g., from local to national) (15).

#### The Participatory Impact Pathway Methodology

The ImpresS methodology is divided into five phases: (i) preparation of the case study; (ii) dialogue with the actors to define hypotheses on the context of the programme and the nature of the impacts during a first participatory workshop; (iii) construction of the narrative of the context and history of the programme and of the impact pathway; (iv) characterization and measurement of the impacts; and (v) validation with the actors during a second participatory workshop (15).

### Study Area

**Figure 2:**
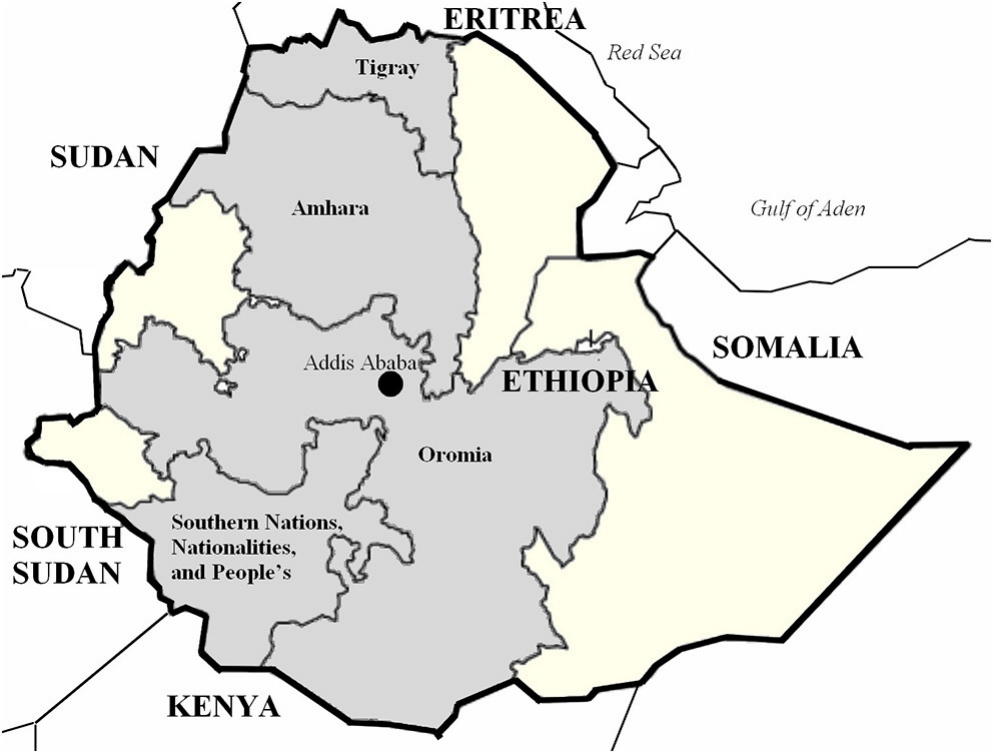
Map of Ethiopia (bold line) and the four regions included in this study (in gray). The capital of Ethiopia, Addis Ababa (black circle), is surrounded by the Oromia region.

This study was conducted in the four regions of Ethiopia where EthioChicken operated in 2018: Tigray, Amhara, Oromia and the Southern Nations, Nationalities, and People's region (Figure 2). The four regions are among the most populated regions in Ethiopia, accounting for more than 80% of the Ethiopian population. Those four regions accounted for 95.3% of the total poultry population in 2018 (22) with 31.8% coming from EthioChicken. In 2018, the poultry production of EthioChicken was highest in the region Southern Nations, Nationalities, and People's (37%), followed by Oromia (31%) (Supplementary Table 1).

### Methodology and Research Tools Used for this Case Study

As the program evaluated was a PPP in the veterinary domain (and not a research programme), and as the PPP evaluated was still active and we wanted to provide recommendations to improve the PPP, we adapted the ImpresS methodology (remaining close to it). Our methodology was divided into six steps:

*Step 1*. Preparation of the case study with key PPP actors from public Veterinary Services and EthioChicken managers by identifying the actors to be involved.
*Step 2*. Dialogue with the actors to map the actors directly or indirectly involved or impacted by the PPP, to identify the elements of the context and the history of the PPP; to identify the different inputs, outputs, outcomes or impacts of the PPP; and to identify the potential limits of the PPP.
*Step 3*. Co-construction of the mapping of the actors, the narrative of the context and history of the PPP, and the impact pathway. Discussion of the added value of the PPP to reach these impacts.
*Step 4*. Co-selection of the limits of the PPP that can be improved and co-construction of the improvement scenarios.
*Step 5*. Validation of the final results and co-construction of the final recommendations.
*Step 6*. Measurement of impacts identified based on gray literature, and internal data from EthioChicken.

This methodology used different participatory tools such as individual semi-structured interviews or grouped semi-structured interviews (=focus group), workshops, depending on the results the research team expected, the resources available, and the availability of the actors (23).

For step 2 “dialogue with the actors”, semi-structured interviews, following a previously prepared checklist, were conducted in the four regions. These were mainly individual interviews to facilitate the capture of individual points of view (24). Due to the time constraint, two semi-structured interviews were conducted in groups (focus group discussions) in two regions. The focus groups may obscure individual opinions, but in order to favor consensually validated information, we homogenized the two groups of actors (one group of four growers of 45-day-old chickens, and one group of eight smallholder farmers). Two different checklists were prepared: one for the actors at the conception of the PPP and one for the other actors. The themes covered by the checklist for the actors at the conception of the PPP were: (i) building of the PPP (inputs), (ii) functioning of the PPP (structure, governance, collaboration), (iii) outputs of the PPP. The themes covered by the other checklists were: (i) poultry production, (ii) involvement in the PPP and the EthioChicken model, (iii) interaction with other stakeholders, (iv) benefits of the PPP, and (v) limits of the PPP and scenario of improvement (Supplementary Material 1). Furthermore, two proportional piling exercises were conducted with two groups of actors following the focus groups discussions. The proportional piling is a semi-quantitative method that classifies elements by stacking small objects (such as seeds) on circles, representing the different elements to classify (24). In this case, the elements to be classified were the benefits brought by getting involved in this model of poultry production.

For each of steps 3, 4, and 5, a workshop was organized (three workshops in total). The main goal of these three workshops was to construct the different elements of the evaluation and the recommendations in a collaborative manner. Unlike the focus groups, which were held with homogeneous groups of actors, the workshops should involve the representatives of the different groups of actors directly or indirectly involved in the PPP as well as the representatives of the actors impacted by the PPP: public and private, national and local actors. For each workshop, a maximum of 50 persons was tolerated in order to conduct group work and allow participants to express themselves (according to the facilitation skills in the team, we were able to divide the participants into three working groups per workshop). The goal of the first workshop, conducted during step 3, was to present, improve, and validate the results obtained during step 2, based on the drafts prepared by the research team, regarding: (i) mapping of actors, (ii) elements of the context and the history of the PPP, and (iii) the impact pathway. The goal of the second workshop, conducted during step 4, was to explore the limits of the PPP between EthioChicken and the Ethiopian government and to co-construct improvement scenarios. For the discussion of limits and improvement of the PPP evaluated, in this second workshop, a wide range of actors, including potential opponents, was wanted. The goal of the third workshop, conducted during step 5, was to present and validate the final report with the actors directly involved in the PPP.

For step 6 “measurement of impacts”, the results of the previous steps were used, as well as gray literature and internal data from EthioChicken such as company profile, and results of their client surveys.

### Period, Target Population, and Sampling Strategy

#### Period

The first field investigation including individual and grouped interviews, proportional piling, and the first two workshops was conducted between March and June 2018. The measurement of impacts was done from September to December 2018. The third workshop was conducted in August 2019.

#### Target Population

Participants should represent a variety of stakeholders from national and local levels directly or indirectly involved in the PPPs between EthioChicken and the public Veterinary Services. Participants should correspond to public and private partners involved in the PPP, actors who influence the PPP, or actors impacted by the PPP. Defining the target population was an iterative process. As we moved forward with mapping of the actors, we identified new categories of actors to include in the participatory evaluation. We sought to include grower agents representative of this category, i.e., 30% women and with flocks of 1,300 chicks per cycle time on average (the numbers do not differ significantly between the four regions). We also sought to include smallholder farmers representative of this category, i.e., 90% of women raising 5–40 chickens on average (the numbers do not differ significantly between the four regions). Actors from almost every category of the target population were interviewed (see the Results Section Mapping of the Actors and Participants Involved in This Study and Supplementary Table 2 presenting the participants of this study).

#### Sampling Strategy

The main goal was to capture a diversity of points of view, representing the different categories of actors in the target population. First, individual semi-structured interviews were conducted at the national level with actors at the conception of the PPP. Then, in the four regions, areas where grower agents operate and villages where smallholders' farmers buy chickens from grower agents were selected. The first list of participants was composed of purposively selected actors, thanks to the help of the EthioChicken manager and village leaders. Then, a non-probability snowballing sampling was used in the four regions, and the initial participants' list was enlarged through the identification, by participants, of other actors who could be included in the study (25). The number of interviews for each category of actors was determined by adapting the concept of saturation. Saturation in a category of actors was considered to be reached when additional interviews provided no new information compared to previous interviews (26). The sample size was therefore not predefined. However, given the time and resource constraints, certain categories of actors were privileged to reach this level of saturation. These categories included actors at the conception of the PPP (actors from EthioChicken, actors from the public Veterinary Services, other actors from the Ministry of Livestock and Fisheries) and actors who adopted the PPP model (growers of 45-day-old chickens, also called grower agents, and smallholder farmers).

### Data Collection

#### Individual and Grouped Semi-structured Interviews

The individual semi-structured interviews lasted from 20 to 30 min. The two focus group discussions lasted 45 min and 1 h. Individual semi-structured interviews and focus group discussions were performed by teams of one Ivorian male researcher (**BN'g**), one Ethiopian male sales manager at EthioChicken (**FT**), three male staff of EthioChicken, and one Ethiopian male veterinarian. All had a veterinary medicine or epidemiology degree and were previously trained in participatory approaches. Only the regional sales members had a relationship with the interviewees as part of their activities. The interviews were conducted in English or local languages (Amharic, Oromifa, Tigrinya, and Wolaytinya), depending on the interviewee. All the discussions were recorded once the interviewee had agreed to participate in the study and be recorded.

#### Proportional Piling

These exercises were done right after each of the two focus groups (**BN'g** and **FT**). Circles were drawn on a large white sheet of paper, representing the benefits mentioned during the two previous focus group discussions. For the group of growers of 45-day-old chickens, three circles were drawn as three benefits were mentioned (“better life”, “job opportunity”, “low investment in terms of land and capital”). For the group of four smallholder farmers, four circles were drawn as four benefits were mentioned (“women's empowerment”, “profit”, “easy to manage”, “low investment in terms of land and capital”). Then, 100 beans were given to each group and the actors were asked to stack the beans. The more the benefit was important to them, the more beans they had to put in. Once the distribution of beans among the different benefits was completed, the research team counted the beans, recorded the scores in percentage (e.g., if 29 beans were put on the circle “profit” then it was noted “profit is 29% of total benefits perceived”), and took photos.

#### Workshops

Two researchers (**MPe**, a French female veterinarian and **BN'g**, an Ivorian male veterinarian) and four facilitators (**FT**, one Ethiopian male sales manager at EthioChicken, and **YT.A** and two other Ethiopian male researchers from the International Livestock Research Institute) conducted the three participatory workshops. The facilitators were trained to moderate, observe, and take notes during the workshop. One observer took extensive notes (**IDL**). Two different groups were set up for each of the workshop: one for English speakers and the other for Ethiopian (Amharic) speakers. The discussions were conducted in English and Amharic, ensuring that all stakeholders took part in the discussions (27). The three workshops lasted around 4 h each, and extensive notes were taken.

#### Measurement of Impacts

The potential indicators of impacts were identified during the second workshop when constructing the impact pathway. Then, the results of the two proportional piling exercises conducted after the two focus groups with smallholder farmers and growers of 45- day-old chickens, gray literature, and the internal data of EthioChicken were screened to quantify the impacts through indicators (**MPo**). The results from individual and grouped semi-structured interviews were also used to measure the impacts in a qualitative manner (**MPo**).

### Data Processing and Analysis

The recorded discussions (i.e., the individual semi-structured interviews, the two focus group discussions), and the manual notes (taken during individual and grouped semi-structured interviews and during the three workshops), were transcribed into English. A unique number was given to each of the transcripts to ensure the anonymity of the interviewees. The transcripts were read, and themes (represented by codes and subcodes) emerged from the reading, corresponding to the functional process of the PPP (Supplementary Material 2). A spreadsheet containing these codes and subcodes was prepared. During a second reading of the transcripts, the qualitative data were classified in the spreadsheet according to its corresponding themes (code/sub-codes) (28). A second spreadsheet database was prepared to draw the impact pathway, using different categories: inputs, outputs, outcomes, and impacts. During another reading of the transcripts, we classified the data in this second spreadsheet database. The results of the two proportional pilings were documented using photographs and were reported in a Word document.

Workshop results such as drawings and notes were documented using photographs. The notes from the three workshops were faithfully transcribed and classified in the same spreadsheet databasestas for semi-structured interviews. The drawn impact pathway developed during the first workshop was reproduced on the CIRAD Impress tool (https://impress-impact-recherche.cirad.fr/resources/impress-knowledge-management-system).

All the data and recommendations were validated during the third workshop, except the measurement of impacts. The impact measurement results were sent to the actors of the conception of the PPP and discussed through email exchanges.

### Ethics

The approval to implement this participatory evaluation was obtained from the managing director of the EthioChicken and the director of the poultry production department of the Ministry of Livestock and Fisheries. The semi-structured interviews and the workshops were carried out after presenting the study objectives and obtaining verbal consent from all volunteer participants. The interviewees could stop the interview whenever they wished. The names and contact details of interviewees were kept in a secured database that is only accessible to the research team, the privacy rights of participants were fully protected, and all data were anonymized.

## Results

### Mapping of the Actors and Participants Involved in this Study

**Figure 3:**
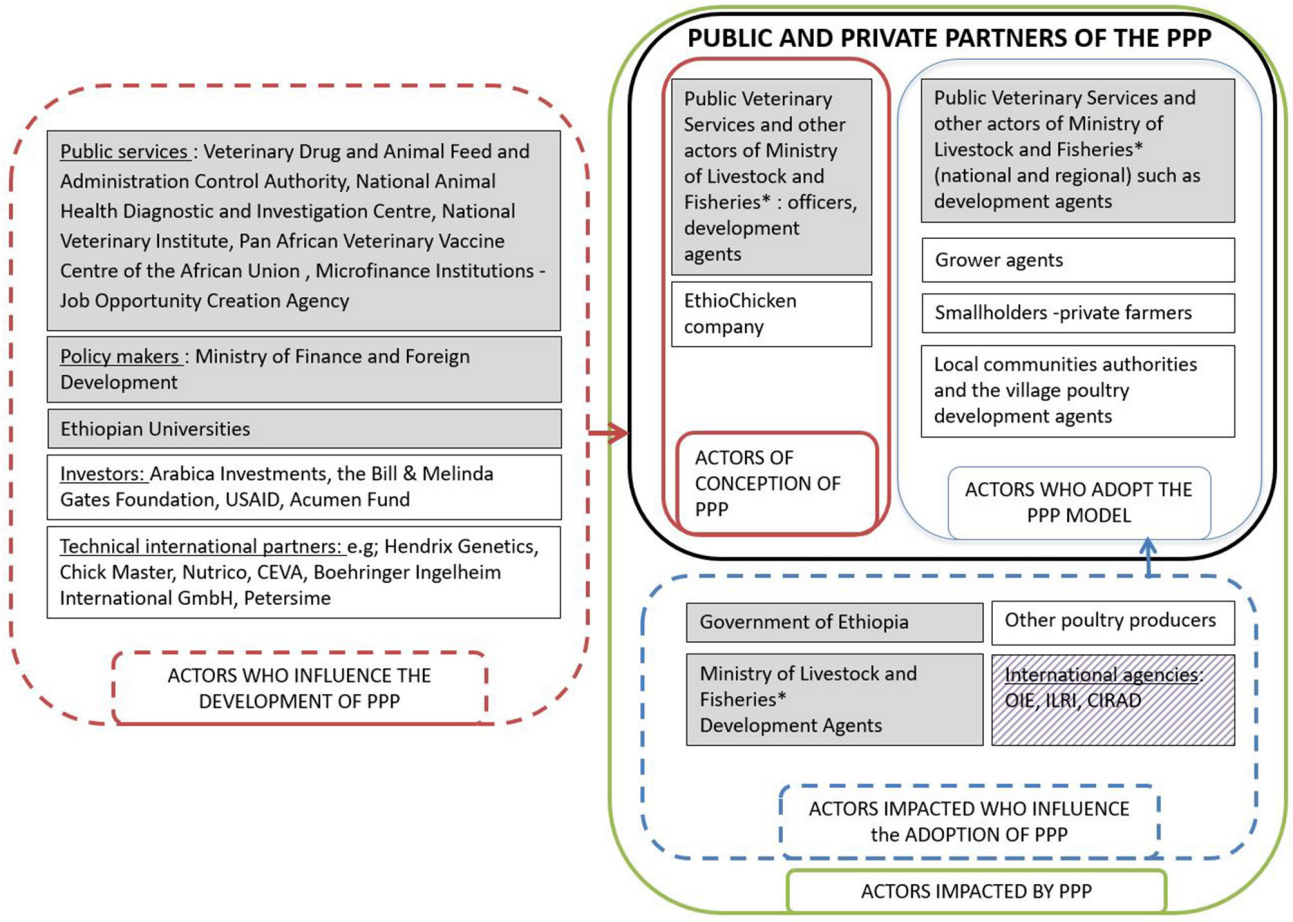
Mapping of categories of the actors involved directly or indirectly in the PPP between EthioChicken and the public Veterinary Services. The dark-gray rectangles indicate the public actors. The white rectangles indicate the private actors. The light-gray rectangle indicates international agencies. *The Ministry of Livestock and Fisheries has merged with Ministry of Agriculture since April 2018. CIRAD, French Agricultural Research Center for International Development; OIE, World Organization for Animal Health; PPP, Public–Private Partnership; USAID, United States Agency for International Development.

Different actor categories were distinguished: actors of conception of the PPP, actors who adopted the PPP model, actors impacted by the PPP and also influencing the adoption, and actors who influence the development of the PPP. The actors can belong to several categories. Actors positively or negatively impacted by the PPP could either be the public and private partners and could also influence the adoption of the PPP model (Figure 3).

The actors who played a major role in conception of the PPP were the public Veterinary Services and other actors of the Ministry of Livestock and Fisheries and EthioChicken company (Figure 3).

The actors who adopted the model on the public side were the public Veterinary Services and other actors of the Ministry of Livestock and Fisheries (livestock officers and public development agents) at regional and national level. The public development agents were public actors who distributed the 45- day-old chickens produced by the grower agents at local level to smallholder farmers. The actors who adopted the model on the private side were grower agents, smallholders' farmers, local communities and, the village poultry development agents (Figure 3). The grower agents (independent private actors) raised day-old chicks supplied by EthioChicken until 45 days, provided poultry healthcare such as vaccination programs, and were assisted by EthioChiken. The village poultry development agents (independent private actors) were actors elected by the local communities to deliver the 45-day-old chickens from the grower agents to the smallholder farmers, operating in two regions due to the non-availability of public development agents.

The actors who influenced the adoption of the PPP model were the government of Ethiopia (public services structures and availability, laws, and regulations), especially the Ministry of Livestock and Fisheries, international agencies, and other poultry producers. The actors who influenced the development of the PPP model (intentionally or unintentionally) did not play a direct role in the conception. On the public side, they were actors of the public services, policymakers, or actors of the Ethiopian universities. On the private side, they were investors or technical international partners (Figure 3).

A total of 64 semi-structured interviews were conducted. Almost all groups of actors identified in the mapping of actors have been included, with the exception of some actors who influenced the development of PPP: investors and technical partners (due to their non-availability on the field, being international actors) and the Ministry of Finance and Foreign Development (due to resource and time constraints) (Supplementary Table 2). Participants were from different administrative levels: international (*n* = 4), national (*n* = 12), regional (*n* = 7), district (*n* = 13), and ward level (*n* = 28). All the interviews at international and national level were given in English, while interviews given at regional, district, and ward level were given in local language. On the 48 interviews conducted at regional, district, and ward level, more interviews were conducted in Oromia (*n* = 19, 39%) and Southern Nations, Nationalities, and People's (*n* = 17, 35%) as the EthioChicken production was higher than in the two other regions (Supplementary Table 2). The actors involved in the interviews represented public (*n* = 20) and private actors (*n* = 44). The individual semi-structured interviews involved 52 participants, while the two focus groups (followed by proportional piling) gathered eight grower agents and four women smallholder farmers. The eight grower agents involved in the focus group were women (25%, *n* = 2) and men (75%, *n* = 6) and possessing flocks of 605 chicks per cycle in average. The 23 smallholder farmers included in individual and group interviews were women (74%, *n* = 17) and men (26%, *n* = 6), and they were raising an average of 27 chickens.

The first workshop had 26 participants, the second 48 participants representing a wide diversity of actors (Supplementary Table 2). The third workshop gathered 18 participants, mainly actors directly involved in the PPP (actors from EthioChiken and actors from public Veterinary Services and other actors from the Ministry of Livestock and Fisheries) (Supplementary Table 2).

### The Context of Implementation of the Public–Private Partnership Between EthioChicken and the Public Veterinary Services: History

**Figure 4:**
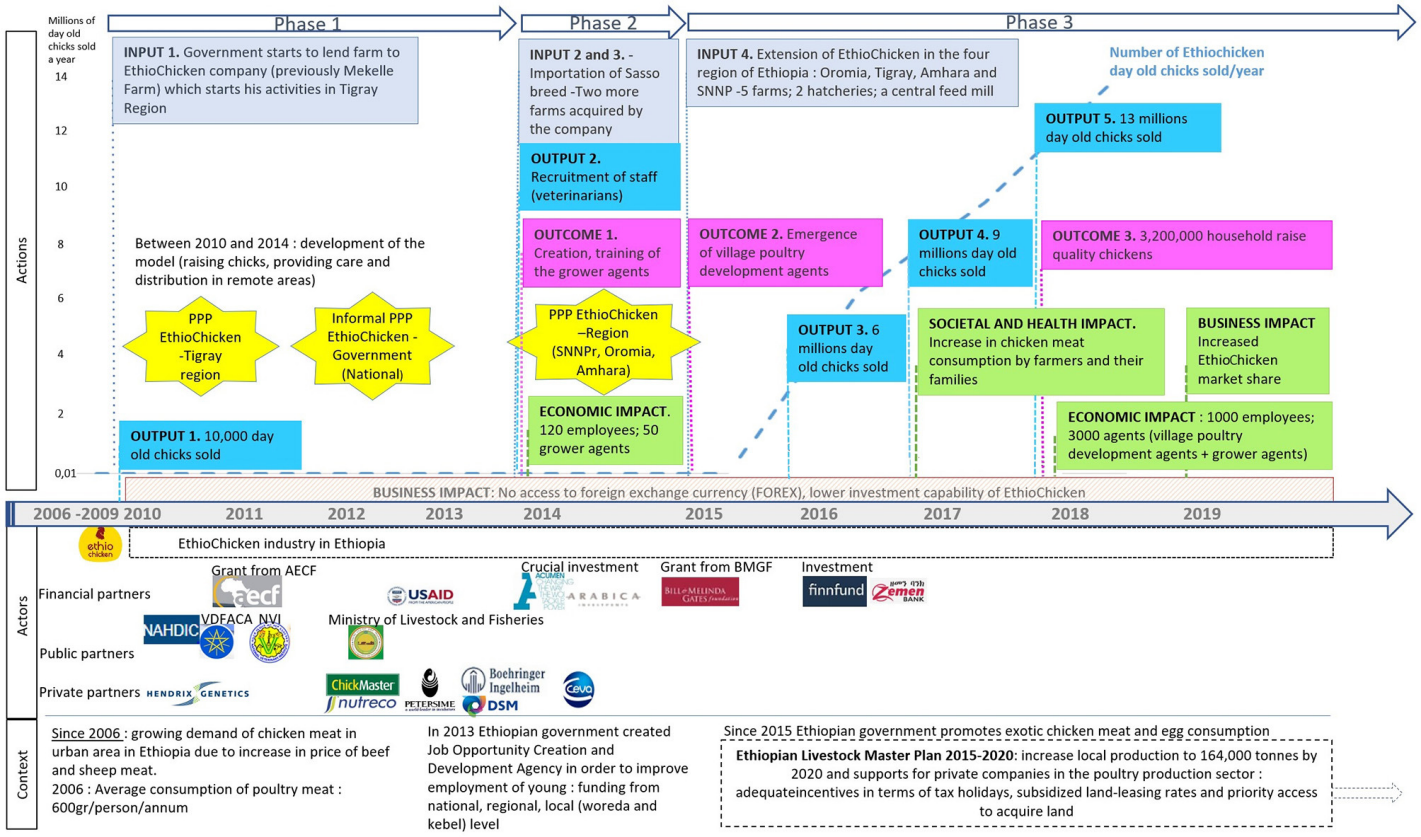
History of the PPP development in three main phases (2010–2019) and impacts; capturing elements of context, actors, and actions. The light-blue rectangles indicate inputs, turquoise ones indicate outputs, pink ones indicate outcomes, and green ones indicate positive impacts and red ones negative impacts. The stars indicate the building of PPPs at national level (second star) and regional level (first and third stars). The actors represented are the financial partners, who have invested in the company EthioChicken, the public partners, and the other private partners. The elements of context are given at the bottom of the figure. The Ministry of Livestock and Fisheries was merged with Ministry of Agriculture since April 2018. AECF, Africa Enterprise Challenge Fund; BMGF, Bill and Melinda Gates Foundation; NAHDIC, National Animal Health Diagnostic and Investigation Center; Forex, Foreign Exchange Currency; NVI, National Veterinary Institute; PPP, Public–Private Partnership; SNNPr, Southern Nations, Nationalities and Peoples' region; VDFACA, Veterinary Drug and Animal Feed and Administration Control Authority; USAID, United States Agency for International Development.

The first phase of the development (2010–2014) of the PPP began in the Tigray region. In 2010, EthioChicken cofounders took charge of a government poultry farm, through an agreement with the Tigray regional government, which was underperforming at that time (*input 1 and first star*, Figure 4). Thanks to the PPP, EthioChicken had access to the extension services of public Veterinary Services of the Ministry of Livestock and Fisheries in Tigray region (first and second stars, Figure 4). Public development agents, public actors from the Ministry of Livestock and Fisheries, distributed chickens at local level to smallholder farmers who could raise them for meat and eggs.

During the second phase of development (2014–2015), the success of the farm in Tigray led the government to recommend that they expand their model to three more regions, thereby expanding the PPP activities (*third star*, Figure 4). EthioChicken started to import dual-purpose improved genetic breed (*input 2*, Figure 4). Since then, the EthioChicken staff have been raising the parental stock (which was imported) and produce day-old chicks in the three regional farms. Grower agents, who were private independent actors contracted by EthioChicken, were created in the four regions to raise the chickens from 1 to 45 days old and to ensure a vaccination program (*outcome 1 and economic impact*, Figure 4). The public development agents continued to deliver the chickens (45 days old) to smallholders' farmers. EthioChicken started to employ young graduate veterinarians from Ethiopian universities (*output 2 and economic impact*, Figure 4).

During the third phase of development (2015–2019), the capacity of EthioChicken expanded into four regions of Ethiopia. Currently, EthioChicken manages five poultry farms (four belonging to the government), two hatcheries, and one feed mill production plant (*input 4*, Figure 4). In two regions, due to the low availability of public development agents, EthioChicken, in agreement with the local communities, has developed village poultry development agents to deliver the 45-day-old chickens from the grower agents to the farmers (*outcome 2*, Figure 4).

During the development of the model, EthioChicken received a crucial investment from different funds and foundations (*financial partners*, Figure 4).

At the time of the study, EthioChicken continued to produce improved-breed day-old chicks, which were distributed to smallholder farmers through the public Veterinary Services network. This model allowed smallholder farmers and their families to increase their consumption of meat (*societal and health impact*, Figure 4). Since 2010, the PPP has increased the number of day-old chicks sold per year (*output 1, 3, 4 and 5*, Figure 4), which were distributed in 2018 to 3.2 million households of smallholder farmers (*outcome 3*, Figure 4). However, the PPP faced important issues linked to access to foreign exchange currency (*business impact*, Figure 4).

### Impact Pathway

#### Inputs

**Figure 5:**
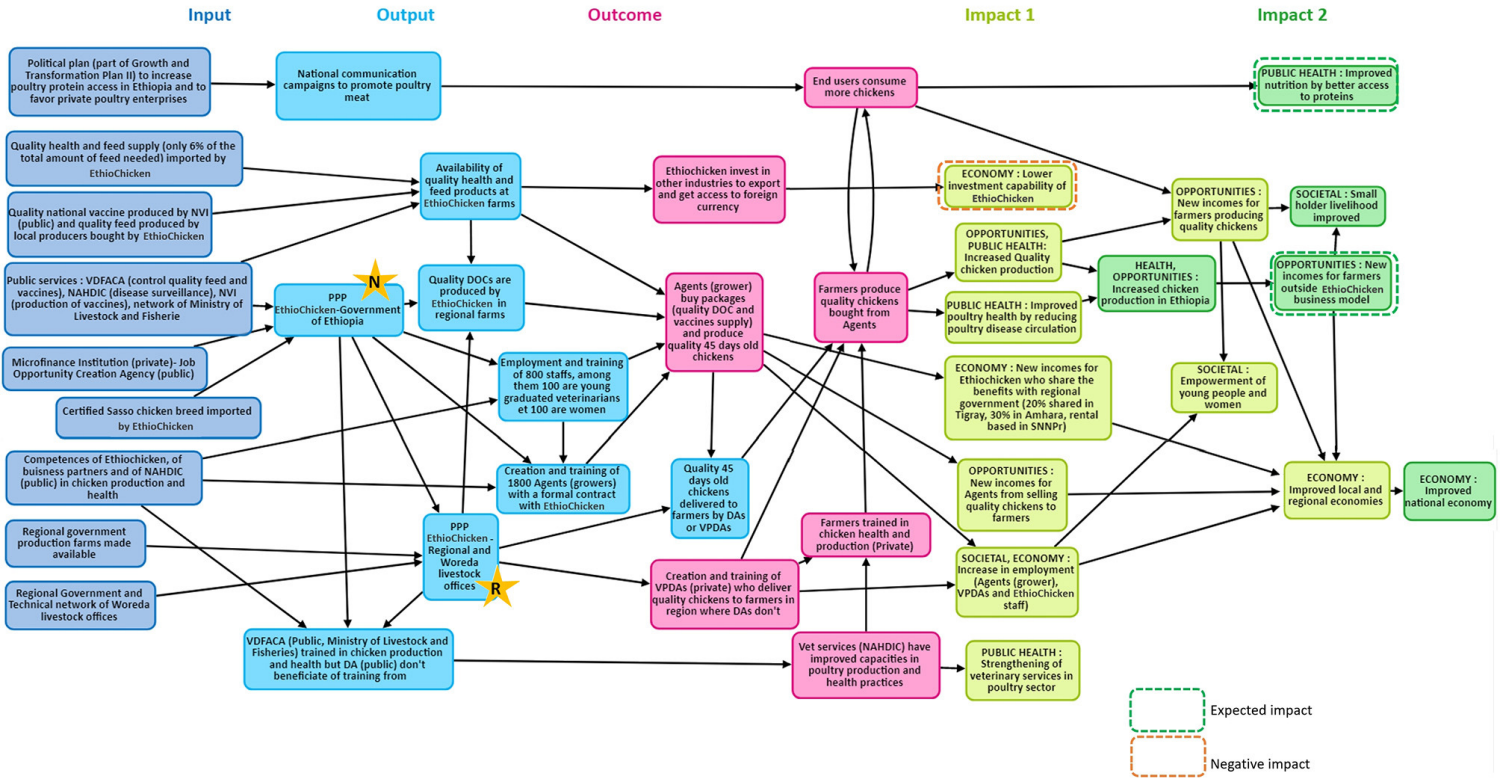
Impact pathway of EthioChicken innovative model and PPP involved in this model: inputs (dark blue), outputs (light blue), PPP at national level (star with N), PPP at regional level (start with R); outcomes (pink), and impacts level 1(light green) and impacts level 2 (dark green). The impacts can be negative (rectangle with dotted red border) or positive (the others). DA, public development agents; DOC, day-old chicks; EC, EthioChicken; NAHDIC, National Animal Health Diagnostic and Investigation Center; NVI, National Veterinary Institute; PANVAC, Pan African Veterinary Vaccine Center of the African Union; VDFACA, Veterinary Drug and Animal Feed and Administration Control Authority; VPDA, Village Poultry Development Agents; Woreda, regions.

The inputs included the political enabling environment: the Growth and Transformation Plan II, and the promotion of exotic chicken meat and egg consumption by the Ministry of Livestock and Fisheries. In 2013, the Ethiopian government created the job opportunity creation and development agency creation, which aims to improve the employment of young people through funding (they can access loans and start to manage a poultry farm) with the collaboration of the private microfinance institution (Figure 5).

The inputs also included (i) public services that provide the authorization of importation and control of the quality of poultry feed and vaccines from other countries, (ii) animal disease surveillance, (iii) the investigation of animal diseases, (iv) the production and control of national vaccines, and (v) extension service network down to ward (kebele) level with technical livestock offices and regional governmental farms (Figure 5).

Other inputs are represented by competencies of EthioChicken and their business partners and public partners in chicken production and health (Figure 5).

Finally, inputs included quality products made available in Ethiopia: improved chicken breeds imported by EthioChicken, quality national vaccines, quality feed produced by local crop producers, quality feed supplies from other countries, and health supplies from abroad. EthioChicken imported two different improved genetic breeds (Sasso and Bonvans breed) from two foreign companies to build up their parental stocks of chickens which they raise in Ethiopia and which produce day old chicks. EthioChicken imported feed from other countries only when the quantity of local feed was insufficient (this accounted for 6% of the total feed purchased by EthioChicken), as well as poultry health supplies (they imported poultry vaccines only when national production was not sufficient). Those inputs were bought in dollars sourced through various means by EthioChicken such as local importers who had access to USD, bank supply agreements and letters of credit from the banks or investor USD (Figure 5).

#### Outputs

National communication campaigns to promote poultry meat were organized by the Ethiopian government. A non-formalized PPP was initiated between EthioChicken and the Government of Ethiopia through the different public actors (Figure 5). Official PPPs, through a memorandum of understanding at regional level started between EthioChicken and regional and district livestock offices. These PPPs conditioned the outputs in terms of employment and training and the production of quality products (Figure 5).

#### Outcomes

The business outcomes included the increased sale of National Veterinary Institute vaccines and of products from local crops, since the demand for vaccines and feed by EthioChicken was high. Grower agents had access to new business with the increased numbers of smallholder farmers willing to buy the 45-day-old chickens produced by EthioChicken genetics (Figure 5).

They were outcomes on employment and training. The creation of village poultry development agents in two regions (where the availability of public development agents was low), to deliver chickens to smallholder farmers, created employment opportunities. These actors were trained in poultry health and management by EthioChicken, and through them and the public development agents, smallholder farmers could receive advices and trainings related to chicken health and production. Actors from the public Veterinary Services (such as the Veterinary Drug and Animal Feed and Administration Control Authority) also received trainings from EthioChicken in poultry production and health practices (Figure 5).

Finally, there were outcomes on the production and consumption of quality poultry products. Thanks to the PPP model, smallholder farmers raised healthy chickens (received at 45 days old) and produced quality eggs and meat, and they and their families consumed more eggs and more chickens (Figure 5). These 45-day-old chickens are produced by private grower agents who purchased day-old chicks from EthioChicken, as well as vaccines. The grower agents managed the vaccination program indicated by EthioChicken. They also received technical assistance from EthioChicken.

#### Impacts

This PPP has led to impacts related to public health, economy, and business (not only at individual but also regional and national levels), as well as societal impacts such as improved education (farmers can send their children to school), women's empowerment, and job employment opportunities (Figure 5, Supplementary Table 3).

##### Economic Impact

There was a positive economic impact on the improvement of local and regional economies due to: (1) the rental of government farms to EthioChicken (20% of the profit from EthioChicken sales goes to the government in one region, and in two regions, EthioChicken paid a monthly rent to use these government farms); (2) the increase of employment with the creation of grower agents who also employed paid staff in order to help them on their farm, the creation of village poultry development agents, and EthioChicken-employed Ethiopian staff; (3) new incomes for many actors due to PPP. There were also second-level economic impacts: increased chicken production in Ethiopia, improved national economy, thanks to improved local and regional economy, and new incomes for farmers outside EthioChicken as this PPP encouraged egg and meat consumption in Ethiopia (Figure 5). Regarding increase poultry production, in 2018, EthioChicken produced 13 million day-old chicks, representing 32.9% of the total chicks and layer hens production in Ethiopia (*n* = 39.4 million).

##### Business Impact

There was a positive business impact for EthioChicken with the new income generated from the sale of day-old chicks to grower agents. There was also a negative business impact on EthioChicken due to the non-availability of foreign exchange currency that threatened EthioChicken activity: they had lower investment capability (Table 1, Figure 5).

**Table 1:** Indicators of business impacts related to different stakeholders generated by the PPPs between the Ethiopian government and EthioChicken.

**Indicator: New incomes**			
**Actors**	**Measure**		**Results**
Farmers	Intensity 1.	Mean annual net benefit per household breeding Sasso chickens	~250 USD^a^^*^
	Intensity 2.	Net benefit (USD) for meat sold per year for flock of 100 heads: EthioChicken breed compared to local breed revenue	**Increase rate: 2.16** EthioChicken breed: 1,017 USD (calculation from^a^) Local breed: 470 USD (calculation from^a^)
	Intensity 2.	Net benefit (Ethiopian Birr) for eggs sold per year for flock of 100 heads: EthioChicken breed compared local breed revenue	**Increase rate: 3.8** EthioChicken breed: 20.5 USD (calculation from^a^) Local breed: 5.4 USD (calculation from^a^)
	Magnitude 3.	% of household that perceived increased income streams after they started rearing chickens from EthioChicken	**74.7%**^b^ (of 3 million household^c^)
Agent	Intensity	Mean annual net benefit per agent for rearing EthioChicken breed	~2,376.84 USD^a^, ^**^
	Magnitude 1.	% of agents who said that profitability is what made the poultry business through EthioChicken stand out from other options	**64%**^a^ (of 3,000,000 household^c^)
	Magnitude 2.	% of agents who perceived that their income had increased since they started this business	**81.4%**^a^ (of 3,000,000 household^c^)

*Intensity reflects the degree of change attributed to the PPP and observed for a given impact, and magnitude reflects the extent or spread of the change*.

a *Internal report made by Research Support Services (Collins O, O., Christopher, C.K., Meseret, M.B., Merihun, N.W.): “Verification study for Africa Enterprise Challenge Fund, Africa agribusiness project: AGFlow poultry' Ethiopia, 2017*.

b *Internal data from EthioChicken: “EthioChicken lean data” Ethiopia, 2016*.

c *Internal data from EthioChicken: “EthioChicken internal statistics” Ethiopia, 2019*.

* *Among the farmers who adopted this PPP model, 79% of households live below 2.50 USD per person per day and 93% reported agriculture as their primary source of income*.

** *In Ethiopia, the average salary per year in 2018 was about 3,652 USD, and the minimum salary was about 495 USD (source: http://www.salaryexplorer.com/salary-survey.php?loc=69&loctype=1)*.

There was a positive business impact for the National Veterinary Institute and national crop producers who sell their products to EthioChicken in large quantities (Table 1, Figure 5).

- “*We have a contract with EthioChicken, in their annual plan they give us a list of vaccines and their quantity, and on this basis we deliver the number of doses. They are developing our business plan because their demand is very high; millions of vaccines are ordered*”. [Interview, head of department of the National Veterinary Institute]

There was a positive business impact for the smallholder farmers and grower agents who produce and sell quality chickens. The four smallholder farmers who participated in proportional piling about the benefits of participating in this PPP model ranked the statement “profit” in second place (representing 29% of the total benefit).

- “*I raise awareness in the communities that buy the chickens, so they are aware how to rear chicken, how to manage and how to benefit from chicken farming”*. [Interview, village poultry development agent]

- “*There is a high demand in credit by young people those days compared to years before, and a huge amount of microfinance institution money has been given to poultry producers* [the grower agent] *which are getting successful. They call their business “printing money” because they get profit in a short time*” [Interview, agent of Microfinance Institution]

##### Societal Impact

The eight grower agents who participated in the proportional piling about the benefits brought by this PPP ranked the statement “better life” in first place (representing 51% of the total benefit) and “job opportunity” in the third place (representing 23% of the total benefit).

-“*[the]Majority of our staff are Ethiopian, we only have two expatriate staff based in Ethiopia […] we are the largest private employers of veterinarians in the country; we contact the Universities in order to interview and nominate students for our training program*”. [Interview, manager of EthioChicken]
-“*We do not have jobs so we want to work, and also chicken rearing can be an optional job*”. [Interview, public development agent]

The four women smallholder farmers who participated in the proportional piling about the benefits brought by this PPP model ranked the statement “women's empowerment” in first place (representing 46% of the total benefit). Women, in most households, were the ones who take care of chicken rearing, and in some households, they were the ones who decided what to do with the revenues from the sale of the eggs and the chickens. EthioChicken had a gender policy in their employment scheme (Table 2, Figure 5).

- “*As women we have to take care of our children and stay at home for our household, and poultry farming doesn't need any huge job so we can do it easily... we can use the money that we earn for ourselves and the kids. Empower women equals empower the community because if the living level of women grows, the community will grow*”. [Discussion during proportional piling, woman smallholder farmer who adopted PPP model]

**Table 2:** Indicators of societal impacts related to different stakeholders generated by the PPPs between the Ethiopian government and EthioChicken.

**Indicators**	**Actors**		**Measure**	**Results**
Direct job created	EthioChicken employees	Magnitude	Number of employees at EthioChicken	1,200^a^
	Qualified EthioChicken employees	Magnitude	Number of veterinarians	100^a^
Indirect job created	Agent	Intensity	Mean salary of agents per year	~2,376.84 USD^*^, ^b^>
		Magnitude	Number of agents	5,000^a^ (among them, only 10% were farmers before^b^)
	Paid staff by the agents	Magnitude	Number of paid staffs by the agents	~4,200 (estimation of 0.84 paid staff/agent^b^)
	Feed crop business	Magnitude	Number of feed companies from which EthioChicken buys crops	82^a^
Satisfaction of improved livelihood	Farmers	Magnitude	% of farmers saying that their life improved since raising EthioChicken chicken	~ 84%^c^
Women's employment opportunities	EthioChicken employees	Magnitude	Number of women employees at EC	400^a^
Women's role in chicken raising	Farmers	Magnitude	% of household with EthioChicken breed where women farmers take care of the chickens	57%^b^
		Magnitude	% of household with EthioChicken breed where women make the main decision on the use of income from chicken products	28.6%^b^

*Intensity reflects the degree of change attributed to the PPP and observed for a given impact, and magnitude reflects the extent or spread of the change*.

a *Internal data from EthioChicken: “EthioChicken internal statistics” Ethiopia, 2019*.

b *Internal report made by Research support services(Collins O, O., Christopher, C.K., Meseret, M.B., Merihun, N.W.): “Verification study for AFRICA ENTERPRISE CHALLENGE FUND Africa agribusiness project: AGFlow poultry” Ethiopia, 2017*.

c *Internal data from EthioChicken. “EthioChicken customer satisfaction survey” Ethiopia, 2017*.

* *NB: In Ethiopia, the average salary per year in 2018 was about 3,652 USD and the minimum salary was about 495 USD (source: http://www.salaryexplorer.com/salary-survey.php?loc=69&loctype=1)*.

Young people were able to create small microenterprises and start their activities as grower agents.

There were also second-level societal impacts: thanks to new incomes, smallholder livelihood was improved and the families were able to send their children to school (Table 2, Figure 5).

- “*I am financially independent and I am fulfilling my house in term of furniture and materials. And I also support my young kid in terms of education tools and money for living expenses*”. [Interview, village poultry development agent]
- “*We want to change our life, from poultry production we profit in terms of money by selling, and we also enjoy meat and egg consumption. […] With a small land and small capital, we can do chicken rearing so we like it*”. [Interview, farmer]

However, there were also farmers who fear to lose their biodiversity of local breed.

-“*There is no consideration in preserving the local genotypes*” [Interview, farmer]

-“[…] *smallholders have preference for the local breeds based on their culture. They are used for adoration of ancestors, or for ceremony to solve disputes*. […]”. [Interview, social scientist in International Livestock Research Institute Ethiopia]

##### Poultry and Public Health Impact

Poultry health was improved by reducing poultry disease circulation due to improved health supplies and health training delivered to grower agents, village poultry development agents, and farmers. Protein intake was improved for smallholder farmers within the PPP model and their families by increased consumption of better-quality chicken products (Table 3, Figure 5).

- “*For us EthioChicken is one of the companies which are contributing to improvement of chicken productivity in Ethiopia*”. [Interview, researcher at International Livestock Research Institute in Ethiopia]

**Table 3:** Indicators of public health impact related to different stakeholders generated by the PPPs between the Ethiopian government and EthioChicken.

**Indicators**	**Actors**		**Measure**	**Results**
Improvement in poultry health management	Agents	Intensity	% of grower agents satisfied with EthioChicken sales manager's advice	84^a^
		Magnitude	% of grower agents who received a visit by the EC sales manager	83^a^
	Farmers	Magnitude	% of farmers confirmed that they had participated in a training organized by EC	21.6^b^
Total meat production by EthioChicken	EthioChicken	Intensity	Increased production meat (tons of kg/year) from 2010 to 2018	From 67.5 to 110,700.0 tons kg/year^c^
		Magnitude	Increased participation of EthioChicken meat out of total meat production in Ethiopia from 2010 to 2018*	From 0.15 to 6.9%^a, d^
Chicken product consumption	Farmer	Intensity 1.	Delta number of EthioChicken and local eggs eaten/week/household	9^a^
		Intensity 2.	Delta number of EthioChicken and local chicks eaten/week/household	3^a^
		Magnitude	Number of households	3,200,000^c^
Meat productivity	Farmers	Intensity	Increased production of meat (ton kg meat/year for flock of 100 heads): EthioChicken breed compared to local breed	47.06 (56.36 – 9.3) (calculation from^e^)
Egg productivity	Farmers	Intensity 1.	Increased number of eggs/years for flock of 100 heads: EthioChicken breed compared to local breed	130 (190 – 60) (calculation from^e^)

*Intensity reflects the degree of change attributed to the PPP and observed for a given impact, and magnitude reflects the extent or spread of the change*.

a *Internal data from EthioChicken. “EthioChicken customer satisfaction survey” Ethiopia, 2017*.

b *Internal data from EthioChicken: “EthioChicken lean data” Ethiopia, 2016*.

c *Internal data from EthioChicken: “EthioChicken internal statistics” Ethiopia, 2019*.

*^d^USDA Foreign agricultural service. Ethiopia's demand for chicken meat is expected to grow. 2017 (accessible here: https://www.fas.usda.gov/data/ethiopia-ethiopias-demand-chicken-meat-expected-grow)*.

e *Internal report made by Research support services: “Verification study for Africa Enterprise Challenge Fund Africa agribusiness project: AGFlow poultry” Ethiopia, 2017*.

The second-level impacts on public health were linked to the strengthening of veterinary services and improved nutrition. Veterinary services were strengthened by the positive impact on poultry health and the increased trust between farmers and veterinary agents (Table 3, Figure 5).

- “*We get some trainings from EthioChicken about important poultry diseases*”. [Interview, staff from the veterinary services, veterinary drug and animal feed and administration control authority]

Improved nutrition through better access to protein was another public health second-level impact. This impact was due to the consumption of improved chicken quality and increased availability of chicken products. A governmental study (an internal communication) showed that the rate of stunting due to malnutrition in infants in the Tigray region decreased from 51% in 2015 to 38% in 2017. This study also showed that the increased of products from chickens raised in rural area and delivered by EthioChicken played an important role in the decrease of the infants' stunting (Table 3, Figure 5).

##### Impact on Trust

Farmers' and consumers' trust in the veterinary services increased, thanks to the improved competencies of veterinary services in poultry health. Consumer trust increased with the quality of the chicken produced within the PPP model. The trust of farmers and other actors to start a low-risk business related to poultry production was increased, thanks to the quality of the chicken produced within the PPP model (Table 4, Figure 5).

- “*So when you walk around, it's common to see rural people rearing improved chickens from EthioChicken; they have 50, 100, or 200 chickens. That was not so easy previously*”. [Interview, regional staff from Ministry of Livestock and Fisheries, Addis Ababa]

**Table 4:** Indicators of impact on trust related to different stakeholders generated by the PPPs between the Ethiopian government and EthioChicken.

**Indicators**	**Actors**		**Measure**	**Results**
Quality chicken	Farmers	Magnitude	% of farmers satisfied with the quality of chicken	91%^a^
Increase demand for the product (2014–2019)	Grower agents	Intensity	Increased number of day-old chicks produced/year by EthioChicken (2014–2019)	10,000–16.4 million^b^
		Magnitude 1	Increased number of grower agents (2014–2019)	100–5,000^b^

*Intensity reflects the degree of change attributed to the PPP and observed for a given impact, and magnitude reflects the extent or spread of the change*.

a *Internal data from EthioChicken. “EthioChicken customer satisfaction survey” Ethiopia, 2017*.

b *Internal data from EthioChicken: “EthioChicken internal statistics” Ethiopia, 2019*.

However, there was also a fear of disease outbreak due to a sense of the fragility of the improved breed compared to the local one.

- “*Talking about disease surveillance, what type of disease can be transported to the farmers because of these improved chickens? I would like a project focus on this aspect. Right now we do not have big problems of disease but disease stays as a biggest challenge; parental stock comes from abroad, so how can we regulate this one more efficiently?*”. [Interview, staff from Pan African veterinary vaccine center of the African union]

### Added Value of the Public–Private Partnership to Reach the Different Impacts

The added value of the PPP to reach the different impacts on poultry sector was mentioned by both public and private partners.

- “*We have good relation with this private company, we work with them very closely. EthioChicken have impact on poultry sector, and also, they encourage other private sectors. […] We want increase poultry production, and EthioChicken are working smoothly, they support our work!*”. [Interview, staff from Ministries of Livestock and Fisheries, regional level, Addis Ababa]
- “*We want to increase the market share of poultry meat (on total livestock meat) from 5 to 30% up to 2030. We have an ambitious plan office, and we want to involve private sectors to achieve our target. […] Private sector give us eggs and day old chicks and increase the poultry production of the country*”. [Interview, staff from Ministries of Livestock and Fisheries, national level]
- “*Without this partnership with the government we wouldn't have this distribution network in place. So definitively, the channel of distribution is the added value. It is the strongest aspect of this relationship. […] We both have a common goal which is to distribute more chicken within Ethiopia*”. [Interview, initiator of EthioChicken]

### Limits of the Public–Private Partnership Model and Improvement Scenarios

Several difficulties and limits of the PPP were mentioned. In Ethiopia, the poultry industry is a recent development. The competency of the public Veterinary Services was limited in the poultry sector because of limited training in poultry science during veterinary studies. The feed and health supplies required for the improved breed of EthioChicken were expensive and difficult to access due to low availability. Finally, the end-consumer market of poultry products was unstable, representing a challenge for the stability of the PPP model. Indeed, this is mainly due to religious and cultural practices in Ethiopia: the existence of different fasting periods, up to 200 days per year, during which a significant part of the population does not consume livestock or poultry products in Ethiopia. During those periods, all the different actors of the PPP are affected by the decline in the sale of chicks or chickens. Improvement scenarios of the PPP and recommendations emerged during the second stakeholder workshops.

#### Issues About Access to Foreign Exchange Currency

During the time of the study, the poultry sector was not a priority for the financial and trade part of the Ethiopian Government and did not have access to foreign exchange currency. There were also difficulties related to access to land; indeed, the government distributed the land depending on their production development priority (not the poultry sector) (Supplementary Table 4).

There was a disconnection between the Ministry of Trade for import permits and the Veterinary Authority, leading to difficulties for the delivery of import permits related to veterinary products. This was a limitation for the public veterinary institute (for the import of reagents for the national production of vaccines and diagnostic kit test supplies from abroad) and for EthioChicken for the import of premix feed, vaccines when local ones are not sufficient, and of improved parental chicken stock (Supplementary Table 4). In 2018, the Ministry of Livestock and Fisheries developed a draft poultry policy to improve the situation.

One solution proposed was to promote the benefits of poultry sector at national and regional levels, so as to encourage the government to put products related to the poultry sector on the list of permitted imports and exports. This would allow an access to foreign exchange currency and access to the export market. Large production companies like EthioChicken can help promote the poultry sector to the government (Supplementary Table 4).

#### Access to Capital for Grower Agents and Farmers

The access to loans and capital for youth employment was limited in terms of the number and amount to be able to start a poultry production activity such as grower agents. Indeed, when grower agents had access to a small amount of financial loan, they had to start with a small number of chicks to raise until chicks were 45 days old and their profit was low. Some of them they were unable to reimburse their credit (Supplementary Table 4).

A solution proposed is consisted of the demonstration of the benefits and the financial requirements for poultry production to loan institutions, in order to convince these institutions to be more inclined to issue credit. Moreover, it would be better to deliver credit directly to young grower agents according to their needs for poultry production: currently, the credits are being lent through youth associations (Supplementary Table 4).

#### Poultry Management

Many farmers reported having limited knowledge about poultry management, and, in some occasions, the local Veterinary Services, through their public development agents, had limited capacity to help them. At the time of the study, the Veterinary Services in Ethiopia had limited numbers of veterinarians who are specializing in poultry management, the veterinary curriculum in universities not focusing on the poultry sector (Supplementary Table 4).

A solution proposed was to improve the knowledge of the local Veterinary Services on poultry health. Specialized veterinarians would be able to support the smallholder farmers. The curriculum of the veterinary degree could incorporate more courses on poultry management, and the international universities and the private poultry sector could help the government in doing so. Also, the government could propose a training in poultry management for the public development agents who are already part of the Veterinary Service. Another solution is that the public development agents could be included in the training given by the coordinator from EthioChicken (currently, only the private village poultry development agents are trained). Finally, another solution could be to have a partnership between veterinary public institutions and private actors like EthioChicken to organize trainings on poultry management in national, regional, and local Veterinary Services down to the village (Supplementary Table 4).

#### Limited Dual Genetics Available in the Country Creating a Competitive Environment

EthioChicken holds the exclusive right to distribute one improved genetic breed (the Sasso breed) in Ethiopia through a contract with a French poultry genetics company, the producers of the breed in question. This exclusive right has led to the stigmatization of EthioChicken by other Ethiopian poultry producers including day-old chicks (from other breeds) for sale to farmers. Because of this stigmatization, EthioChicken did not have access to the association of poultry producers in Ethiopia, limiting its market access. The absence of EthioChicken in the association also decreased the strength of the latter and its lobbying option, EthioChicken being an important factor in poultry production in Ethiopia. The functioning model of other poultry producers was different from EthioChicken's, as they sell chickens at any stage to farmers (not necessarily at 45 days) without the intermediary of grower agents nor the package of vaccines and trainings. This explains the reason that farmers tended to adopt the EthioChicken model compared to other models and become contracted grower agents. This increased the stigmatization of EthioChicken by other Ethiopian poultry producers (Supplementary Table 4).

One solution would be to promote the access of alternative improved genetic to other Ethiopian poultry producers. However, if other poultry producers provide improved genetics without the full model (health, feed supplies and post-sale services and trainings) this could lead to limited improved production. Without the full model, in the long term, the success of other poultry producers could decrease. The solution would be to promote the “transfer” of a similar model (the EthioChicken model) through PPPs to other competitors to guarantee the quality and impact of the actions, as is already the case for two poultry-producing companies (Supplementary Table 4).

## Discussion

The results of this study describe (i) the history; (ii) the complex process of the PPP between EthioChicken and Ethiopian government; and (iv) societal, economic, and health impacts brought by this collaboration. The participatory impact pathway methodology captured the viewpoints of public and private partners of the PPP, actors who influenced it, and actors impacted by it, enabling the transparency of the interests, benefits, and constraints of each actor.

### The Importance of Participatory Impact Evaluation Methodology

The main strength of this study lies in the involvement of different actors in the evaluation process. The participatory approaches allowed the recording of viewpoints from a large number of actors from both the public and private sector, actors influencing the PPP, and factors impacted by the PPP, including vulnerable actors such as young people and women. The importance of capturing the viewpoints of the vulnerable groups to enhance equity in health and wellbeing is enhanced in the protocol for PPP evaluation in the public health of the World Health Organization (29).

Another strength of the impact pathway methodology lies in the integrated evaluation of a PPP in the veterinary domain. Indeed, this methodology enabled evaluation of the context (thanks to the analysis of the history), evaluation of the process (thanks to the mapping of actors, the identification of inputs and outputs), and evaluation of the results (thanks to the identification of outcomes and identifications and measurement of impacts). Until recently, a limited number of studies have evaluated PPPs in the veterinary domain. As the quality of PPP outcomes and impacts will depend on the quality of its process organization, the evaluation frameworks of PPPs in public health advise to describe and analyze PPP mechanism. Elements such as relationships between the two sectors, the financial arrangement, governance structure, and functions of the PPP should be taken into account in the evaluation, in addition to the impacts of the PPP (10, 29). The impact pathway methodology that we mobilized allowed us to look at the context, the process of the PPP and its outcomes and impacts (14, 15). PPPs represent a means to achieve objectives and can be transitional; they need to be adapted to their own context, and there is no best way to manage them (30). This is why it is important to mobilize an evaluative research approach, such as the impact pathway methodology, which seeks to understand the how and why of the results, rather than a normative evaluation approach that would seek to compare the components of the intervention to pre-established standards (31). The evaluation we conducted of both the PPP process and PPP impacts was crucial in order to provide appropriate recommendations on how to improve the PPP.

There is general agreement that PPPs should represent an added value compared to a program that does not involve PPPs. However, difficulties in monitoring the added value of PPPs have been identified. Indeed, comparing the results of a PPP with an existing or modeled “counterfactual”, such as a territory without a PPP or a purely public or purely private alternative, is not an easy task. The multiple factors influencing outcomes, and the marked influence of the context make it almost impossible to perform modeling or find an existing counterfactual (32, 33). The best way to overcome this difficulty is to use participatory approaches and to rely on the opinions of public and private partners and for them to discuss together on this potential added value (34, 35), which is what we did. In order to overcome the difficulty of measuring the added value of a PPP, it was important to focus on understanding the causal relation between the implementation of a PPP and its outcomes and impacts, which is what we did using the impact pathway. The representation of the impact pathway also made it possible to visualize which outcomes (and related impacts) depended directly or indirectly on the PPP and to hypothesize that these outcomes in the current situation, without the PPP, would not have been possible.

### The Importance of Considering the Different Types of Impact

Animal health represents a challenge in terms of public health (36), food safety, socioeconomic stability (37), and interaction with the environment (38, 39). We argue that the sanitary as well as economic, business, social, and environmental impacts of animal health programs implemented *via* PPPs or otherwise must be taken into consideration to promote a sustainable livestock system. The methodology of participatory impact pathway by capturing a diversity of viewpoints allowed to gain a systemic understanding of the PPP evaluated and its contribution to impacts. The positive and negative impacts mentioned by the participants of this study relate to economic, business, and societal aspects (livelihood, women's empowerment, education) and to public health (poultry disease control, strengthening of Veterinary Services, improving nutrition). Our study showed that the outcomes/impacts of this PPP varied and went beyond the sanitary and animal productivity range. For example, it is interesting to note than two other Ethiopian poultry producers have already adopted the same model as EthioChicken (intermediary grower agents who raise and care for the chicks until they are 45 days old and collaboration with public actors for the distribution of chickens) but with other improved genetic breeds, which can potentially provide second-level impacts. Another example is the strengthening of the Veterinary Services, as was captured in this case study through the trainings of the different actors linked to the Veterinary Services in poultry health. Bryson et al. (34) argue that PPP should result in “public value” that could not be created without the PPP. In the veterinary domain, one public value would be the strengthening of the Veterinary Services.

However, we did not investigate further the fear expressed by some farmers of the decrease of their local breeds and of the immune fragility of improved genetic breeds. These elements might have deserved attention. Indeed, the genetic diversity of domesticated animals is also on the list of biodiversity indicators by the European Academies' Science Advisory Council (40) and the loss of livestock biodiversity is raising sustainability issues (41). It is recognized that there is a need to maintain a broader range of animal genetic resources to be able to deal with future uncertainties, such as climate change and zoonotic diseases (42). It is normal for any program to have externalities, consequences not foreseen in the planning, and an implementation of the program. However, the Food and Agriculture Organization proposes to integrate the externalities as of the planning process to achieve a sustainable program (43). Taking account of externalities, by anticipating them and undertaking corrective action of the negative ones, may help the PPP to be stable over time and increase its legitimacy in society. For this case study, the adaptation of this model (which includes training in poultry healthcare and a distribution model to remote areas) to local breeds rather than or in addition to genetically improved breeds could have been discussed in the workshops. This would also avoid a dependence on the imports from other countries of genetically improved poultry.

### Importance of Collaboration at Different Levels and Trust Between Partners

The study showed that the PPP between EthioChicken and the Ethiopian governments takes place at different administrative levels: national and regional. This allows EthioChicken and the State to develop the poultry sector in marginal areas. Indeed, as mentioned by Ahuja (3), in their analysis of the economic rationale of sector roles in the provision of animal health services, which stressed the importance of a division of labor between the public and private sectors, the collaboration between the private and public sector is particularly important to reach remote areas.

We showed that each actor derives his/her own benefit from participating in PPP. However, there are associated constraints, and the participatory workshops allowed the partners to codevelop scenarios to overcome such constraints. The PPP reference guide from the World Bank emphasizes the need to compile a complete and transparent list of risks associated with the PPP and to think about risk allocation (44). The participatory approaches allowed the partners to clearly identify those risks and thus to be able to limit them.

Finally, participatory evaluation has benefits in itself. Involving the different stakeholders during the evaluation brings out the benefits and constraints of different stakeholders, to increase transparency between the partners, thereby increasing trust and collaboration (18). The literature on PPPs in public health emphasizes the need for partners to understand their mutual motivations and objectives (30), and this exchange during the participatory evaluation helped to clarify people's expectations about various aspects of the PPP. Participatory approaches in evaluation have also proven to be very useful in ensuring the adaptability, acceptability, and relevance of the recommendations and therefore ease the implementation of corrective actions (20). Indeed, actors can share their perception of the PPP and codesign the corrective actions needed to ensure the reach of expected impacts (15). The different workshops with the various stakeholders facilitated a reflection and analysis of the system in which they are involved.

### The Difficulty to Differentiate Outcomes and Impacts

The difference between outcomes and impacts is not easy to determine. The impacts are what remains after the project is completed. In the literature on the evaluation of PPP in the public health and veterinary domain, the difference between outcomes and impacts was established in only one reference (45). The framework of the Centers for Disease Control and Prevention (10) proposed to write the logic model of the partnership by collecting information on a partnership's inputs, activities, outputs, outcomes, and impacts and by linking these different elements together, which has been done during this study. However, no further information was given to differentiate the outcomes and impacts. In this case study, this difficulty was accentuated by the fact that our evaluation was made “*in itinere*”, as the PPP was not over. So, to be sure that what we called impacts correspond to the long-term results of the PPP, an ex-post evaluation should be done to analyze what remains after the PPP is over (as the PPP can be transitional).

### Limitations

We are aware that some results might have been distorted by several factors and should then be interpreted with caution. The translation of the different records is the first possible limitation, as this may have introduced a certain misinterpretation of opinions. Another limitation of the participatory approach is the subjective form of the method, as it depends on the stakeholders' willingness to respond to questions and interact with researchers (46). Stakeholders belonging to the same category may express divergent opinions, and therefore, several stakeholders should be included in the interviews. Due to time constraints, we may not have succeeded in reaching the saturation level for each category of stakeholder (such as actors who influence the development or adoption of the PPP). However, for the actors at the conception of the PPP and the actors who adopted the PPP, we are confident in saying that we have reached a saturation level. The grower agents included in this study were representative in terms of the proportion of women (25%), and though the average flock size per cycle (*n* = 605) was lower than the average for this category (*n* = 1,300), this is unlikely to have influenced the results obtained. Due to time and resource constraints, the grower agents involved were all from the same region (Oromia). Ideally, grower agents should have been from the four different regions, but as the system is the same in all four regions for this category of actors, this is unlikely to have influenced the results obtained. The smallholder farmers included in this study were representative in terms of the proportion of women (74%) and the average number of chickens raised (*n* = 27).

Another limit of our study relies on the fact that participatory approaches cannot erase pre-existing social conditions that may hamper the capacity of actors to express themselves freely. Representing the diversity of viewpoints from stakeholders who influence, are involved in, or impacted by the PPP during the evaluation process was a challenge. The genuine participation of all stakeholders may not have been fully achieved, especially during the workshops, since power structures limit the free expression of marginalized people (47). However, we believe that the creation of several small groups during the workshops, and the conducting of several individual interviews, limited this self-censorship. Women play an important role in rural areas and especially in poultry raising. We paid attention to respecting the ratio of women for the grower agents and for smallholders during the semi-structured interviews in order to hear their voices. However, the researchers that interviewed them were male, which could have influenced their responses, although they were careful to limit this bias (one of the researchers was Ethiopian and was careful to respect cultural practices).

### Application and Perspective

This study allowed us to provide recommendations at policy level. Indeed, the Ministry of Livestock and Fisheries and the Ministry of Finance and Economic Development were present during the workshop. The recommendations are related, for example, (i) to foreign exchange currency access for stakeholders involved in poultry production, (ii) to the need for training in poultry production to be included in the veterinary curriculum, and (iii) to the increase of access to loans to young agents or farmers for the start of a poultry business.

The results of this evaluation, together with other documents and in collaboration with stakeholders involved in PPPs worldwide, were used to develop the *OIE PPP Handbook* (2) in order to provide a model that could potentially be scaled-up in other countries, when and if relevant, to be able to improve the performance of Veterinary Services.

This represent an in-depth case study, which can contribute to the scientific discipline of evaluation applied to PPPs in the veterinary domain. This case study represents an in-depth analysis of a PPP corresponding to the cluster 3 “transformative” category in the typology from Galière and al. (4). It would be interesting to have other case studies related to PPPs in cluster 1 “transactional” and cluster 2 “collaborative”.

## Conclusion

The diverse impacts (economic, business, society, and health) linked to the poultry sectors identified in this study have been made possible by PPPs at the different administrative levels of the country. Further work should be done on PPPs in the veterinary domain to better characterize the respective responsibilities, risks, and benefits for each actor involved. Indeed, PPPs in the veterinary domain are spread all over the world and are often complex, dynamic, multilevel systems. The constraints and limits identified during this study require strong communication between public and private actors from different sectors, to be solved. This impact pathway methodology, based on participatory evaluation, applied for the first time in the evaluation of a PPP in the veterinary domain, helped to formulate recommendations to improve PPPs. This case study provides the context-dependent evaluation outputs of a PPP related to cluster 3 “transformative” and represents a milestone in building an evaluation framework of PPP in the veterinary domain.

## Data Availability Statement

The raw data supporting the conclusions of this article will be made available by the authors, without undue reservation.

## Ethics Statement

Ethical review and approval was not required for the study on human participants in accordance with the local legislation and institutional requirements. Written informed consent for participation was not required for this study in accordance with the national legislation and the institutional requirements.

## Author Contributions

N'gN'G designed the methodology and the study protocol and realized the field investigation. MPo provided hypothesis for research, analyzed the data, wrote the first version of the manuscript, and integrated co-authors' inputs. ID-L was in charge of the funding acquisition and supervised the study. YA, BW, FT, and UD organized and supervised the field investigation. PT designed the methodology and the study protocol. MPe provided hypothesis for research, designed the methodology and the study protocol, and supervised the study. All authors of the manuscript significantly contributed to the revision, read, and approved the submitted version.

## Funding

This study was carried out as part of the OIE Public Private Progress Project, in collaboration with CIRAD, supported by the Bill & Melinda Gates Foundation under grant number: OPP1159705.

## Conflict of Interest

FT and UD were employed by Ethiochicken at the time of the study. The remaining authors declare that the research was conducted in the absence of any commercial or financial relationships that could be construed as a potential conflict of interest.

## Publisher's Note

All claims expressed in this article are solely those of the authors and do not necessarily represent those of their affiliated organizations, or those of the publisher, the editors and the reviewers. Any product that may be evaluated in this article, or claim that may be made by its manufacturer, is not guaranteed or endorsed by the publisher.

## Footnotes

^1^As defined in article 3.4.2 of the *Terrestrial Animal Health Code*. of the OIE (https://www.oie.int/index.php?id=169&L=0&htmfile=chapitre_vet_legislation.htm): “Veterinary domain means all the activities that are directly or indirectly related to animals, their products and by-products, which help to protect, maintain and improve the health and welfare of humans, including by means of the protection of animal health and animal welfare, and food safety”.

^2^As defined in the glossary of *Terrestrial Animal Health Code*. of the OIE, “Veterinary Services means the governmental and non-governmental organisations that implement animal health and *welfare* measures and other standards and recommendations in the *Terrestrial Code* and the OIE *Aquatic Animal Health Code* in the territory”.

^3^The Ministry of Livestock and Fisheries has merged with the Ministry of Agriculture since April 2018.

## References

[1] World Organisation for Animal Health. Public–private partnership and perspectives in the veterinary domain. Bulletin de l'OIE. (2020) 2019:1–2. 10.20506/bull.2019.2.2973

[2] World Organisation for Animal Health. The OIE PPP Handbook: Guidelines for Public-Private Partnerships in the Veterinary Domain. (2019). Available online at: https://www.oie.int/publicprivatepartnerships/ppp/en/Handbook_en.html (accessed March 11, 2020).

[3] Ahuja V. The economic rationale of public and private sector roles in the provision of animal health services. Rev Sci Tech. (2004) 23:33–45. 10.20506/rst.23.1.1464 15200085

[4] Galière M Peyre M Muñoz F Poupaud M Dehove A Roger F. Typological analysis of public-private partnerships in the veterinary domain. PLoS ONE. (2019) 14:e0224079. 10.1371/journal.pone.0224079 31671123 PMC6822735

[5] Trading Economics,. Ethiopia Rural Population. Trading Economics (2019). Available online at: https://tradingeconomics.com/ethiopia/population (accessed May 2, 2019).

[6] Central Statistical Agency of Federal Democratic Republic of Ethiopia. Agricultural Sample Survey 2020/21 [2013 E.C.], Volume II, Report on Livestock and Livestock Characteristics. (2021). Available online at: https://www.statsethiopia.gov.et/wp-content/uploads/2021/05/REVISED_2013.LIVESTOCK-REPORT.FINAL-1.pdf (accessed November 2, 2021).

[7] USDA Foreign agricultural service,. Ethiopia's Demand for Chicken Meat Is Expected to Grow. Global Agricultural Information Network (2017). Available online at: https://www.fas.usda.gov/data/ethiopia-ethiopias-demand-chicken-meat-expected-grow (accessed July 18, 2018).

[8] Ministry Ministry of Agriculture Livestock Resources Development, Sector,. Ethiopia Livestock Master Plan. Roadmaps for Growth and Transformation - a Contribution to the Growth and Transformation Plan II (2015-2020). (2015). Available online at: https://www.researchgate.net/publication/283781705_Ethiopia_livestock_master_plan_Roadmaps_for_growth_and_transformation (accessed November 1, 2021).

[9] Allen W,. Planning, Monitoring Evaluation - Closing the Loop. Learning for Sustainability. (2019). Available online at: https://learningforsustainability.net/plan-monitor-evaluate/ (accessed April 22, 2020).

[10] Rieker P,. Partnership Evaluation: Guidebook Resources. Centers for Disease Control Prevention (CDCP), Division of Nutrition, Physical Activity, Obesity. (2011). Available online at: https://www.cdc.gov/obesity/downloads/PartnershipEvaluation.pdf (accessed March 11, 2021).

[11] Rushton J. Animal health economics : where have we come from and where do we go next? CAB Rev. (2007) 2:72031. 10.1079/PAVSNNR2007203127049255

[12] Hendrikx P Gay E Chazel M Moutou F Danan C Richomme C. an assessment tool of epidemiological surveillance systems in animal health and food safety. Epidemiol Infect. (2011) 139:1486–96. 10.1017/S0950268811000161 21385516

[13] Delabouglise A Dao TH Truong DB Nguyen TT Nguyen NTX Duboz R. When private actors matter: Information-sharing network and surveillance of Highly Pathogenic Avian Influenza in Vietnam. Acta Trop. (2015) 147:38–44. 10.1016/j.actatropica.2015.03.025 25847263

[14] Douthwaite B Kuby T van de Fliert E Schulz S. Impact pathway evaluation: an approach for achieving and attributing impact in complex systems. Agric Syst. (2003) 78:243–65. 10.1016/S0308-521X(03)00128-8

[15] Barret D Blundo Canto G Dabat M-H Devaux-Spatarakis A Faure G Hainzelin E. Impress methodological guide to ex post impact of agricultural research in developing countries. Cirad. (2018) 2018:6. 10.19182/agritrop/00006

[16] de Janvry A, Dustan, A, Sadoulet, E,. Recent Advances in Impact Analysis Methods for Ex-post Impact Assessments of Agricultural Technology: Options for the CGIAR. University of California at Berkeley (2010). Available online at: https://cas.cgiar.org/sites/default/files/images/deJanvryetal2010_0.pdf (accessed November 1, 2021).

[17] Springer-Heinze A Hartwich F Henderson JS Horton D Minde I. Impact pathway analysis: an approach to strengthening the impact orientation of agricultural research. Agric Syst. (2003) 78:267–85. 10.1016/S0308-521X(03)00129-X

[18] BetterEvaluation. Participatory Evaluation. (2012). Available online at: https://www.betterevaluation.org/en/plan/approach/participatory_evaluation (accessed February 4, 2021).

[19] Bryson JM Patton MQ Bowman RA. Working with evaluation stakeholders: a rationale, step-wise approach and toolkit. Eval Program Plann. (2011) 34:1–12. 10.1016/j.evalprogplan.2010.07.001 20674980

[20] Calba C Antoine-Moussiaux N Charrier F Hendrikx P Saegerman C Peyre M. Applying participatory approaches in the evaluation of surveillance systems: a pilot study on African swine fever surveillance in Corsica. Prev Vet Med. (2015) 122:389–98. 10.1016/j.prevetmed.2015.10.001 26489602

[21] Taut S Brauns D. Resistance to evaluation: a psychological perspective. Evaluation. (2003) 9:247–64. 10.1177/13563890030093002

[22] Central Statistical Agency of Federal Democratic Republic of Ethiopia. Agricultural Sample Survey 2017/18 [2013 E.C.], Volume II, Report on Livestock and Livestock Characteristics. (2018). Available online at: https://www.statsethiopia.gov.et/wp-content/uploads/2020/02/Agricultural-Sample-Survey-Livestock-Poultry-and-Beehives.pdf (accessed November 2, 2021).

[23] Alders RG Ali SN Ameri AA Bagnol B Cooper TL Gozali A. Participatory epidemiology: principles, practice, utility, and lessons learnt. Front Vet Sci. (2020) 7:876. 10.3389/fvets.2020.532763 33330678 PMC7672004

[24] Mariner JC, Paskin, R,. Manual on Participatory Epidemiology: Methods for the Collection of Action-Oriented Epidemiological Intelligence. Rome: Food Agriculture Organization of the United Nations (2000). Available online at: http://www.fao.org/3/X8833E/X8833E00.htm (accessed March 20, 2021).

[25] Sadler GR Lee H-C Lim RS-H Fullerton J. Recruitment of hard-to-reach population subgroups *via* adaptations of the snowball sampling strategy. Nurs Health Sci. (2010) 12:369–74. 10.1111/j.1442-2018.2010.00541.x 20727089 PMC3222300

[26] Fusch PI Ness LR. Are we there yet? Data saturation in qualitative research. In: The Qualitative Report NSU Library. Florida. p. 1408–16. Available online at: https://cpb-us-e1.wpmucdn.com/sites.nova.edu/dist/a/4/files/2015/09/fusch1.pdf (accessed January 4, 2022).

[27] Glenn JC,. 3.0 futures research methodology: Millenium project. In: Glenn JC, Gordon TJ, editors. Futures Research Methodology Version. The Millennium Project. (2009). Available online at: https://www.researchgate.net/publication/242703610_Participatory_methods

[28] Campenhoudt LV Marquet J Quivy R. Analyse de contenu. In: Quivy R, Campenhoudt LV, editors. Manuel de recherche en sciences sociales. Paris: Dunod. p. 298–30.

[29] Barr DA. A research protocol to evaluate the effectiveness of public–private partnerships as a means to improve health and welfare systems worldwide. Am J Public Health. (2007) 97:19–25. 10.2105/AJPH.2005.075614 17138922 PMC1716250

[30] National Academies of Sciences. The Role of Public-Private Partnerships in Health Systems Strengthening: Workshop Summary. Washington, DC. p. 126. 27386614

[31] Champagne F Contandriopoulos A-P Brousselle A Hartz Z Denis J-L. L'évaluation dans la santé: concept et méthode. In: Les Presses de l'Université de Montréal. Montréal. p. 35–56. Available online at: http://site.ebrary.com/id/10442484?ppg=34 (accessed January 4, 2022).

[32] Vrangbæk K. Public–private partnerships in the health sector: the Danish experience. Health Econ Pol Law. (2008) 3:141–63. 10.1017/S1744133108004465 18634625

[33] Barlow J Roehrich J Wright S. Europe sees mixed results from public-private partnerships for building and managing health care facilities and services. Health Aff. (2013) 32:146–54. 10.1377/hlthaff.2011.1223 23297282

[34] Bryson JM Crosby BC Stone MM. Designing and implementing cross-sector collaborations: needed and challenging. Public Adm Rev. (2015) 75:647–63. 10.1111/puar.12432

[35] Kamya C Shearer J Asiimwe G Carnahan E Salisbury N Waiswa P. Evaluating global health partnerships: a case study of a Gavi HPV vaccine application process in Uganda. Int J Health Pol Manag. (2016) 6:327–38. 10.15171/ijhpm.2016.137 28812825 PMC5458794

[36] Jones KE Patel NG Levy MA Storeygard A Balk D Gittleman JL. Global trends in emerging infectious diseases. Nature. (2008) 451:990–3. 10.1038/nature06536 18288193 PMC5960580

[37] The High Level Panel of Experts on Food Security and Nutrition. Sustainable Agricultural Development for Food Security and Nutrition: What Roles for Livestock? (2016). p. 140. Available online at: https://www.unscn.org/uploads/web/news/HLPE-Report-10-EN.pdf

[38] Steinfeld H Gerber P Wassenaar T Castel V Rosales M de Haan C. Livestock's long shadow. Environ Issues Options. (2006). Available online at: http://www.fao.org/3/x5305e/x5305e00.htm

[39] Dumont B Ryschawy J Duru M Benoit M Chatellier V Delaby L. Associations among goods, impacts and ecosystem services provided by livestock farming. Animal. (2019) 13:1773–84. 10.1017/S1751731118002586 30333070 PMC6639720

[40] European Academies' Science Advisory Council. A Users' Guide to Biodiversity Indicators. (2004). Available online at: https://royalsociety.org/~/media/Royal_Society_Content/policy/publications/2005/9667.pdf (accessed March 30, 2021).

[41] Tisdell C. Socioeconomic causes of loss of animal genetic diversity: analysis and assessment. Ecol Econ. (2003) 45:365–76. 10.1016/S0921-8009(03)00091-0

[42] Seré C van der Zijpp A Persley G Rege E. Dynamics of livestock production systems, drivers of change and prospects for animal genetic resources. AGRI. (2008) 42:3–24. 10.1017/S1014233900002510

[43] Neven D,. Developing Sustainable Food Value Chains. Guiding Principles. Rome: Food Agriculture Organization of the United Nations (2014). Available online at: http://www.fao.org/3/I3953E/i3953e.pdf (accessed March 11, 2021).

[44] World Bank Institute. Public-Private Partnerships Reference Guide. World Bank Group (2017). Available online at: https://ppp.worldbank.org/public-private-partnership/library/ppp-reference-guide-3-0 (accessed March 11, 2021).

[45] Poupaud M Galière M Dieuzy-Labaye I Antoine-Moussiaux N Peyre M. Toward a framework for the evaluation of public-private partnerships in the veterinary domain: a scoping review. Under publication.

[46] Schmeer K,. Stakeholder Analysis Guidelines. Policy Toolkit for Strengthening Health Sector Reform. (1999). p. 48. Available online at: https://www.academia.edu/28157521/Stakeholder_Analysis_Guidelines

[47] Cooke B. The social psychological limits of participation. In: Cooke B, Kothari U, editors. Participation: the New Tyranny? London: Zed Books. p. 102–21.

