# Supplementary material for: Evaluation of Public–Private Partnership in the Veterinary Domain Using Impact Pathway Methodology: In-depth Case Study in the Poultry Sector in Ethiopia
Source: Front Vet Sci. 2022 Feb 22;9:735269. doi: 10.3389/fvets.2022.735269 (PMC8901995; doi:10.3389/fvets.2022.735269)
Supplement: Supplementary file 1 [file Table_1.DOCX]

**Supplementary Table 1.** Poultry population in Ethiopia, per region, and produced by EthioChicken in 2018.

|  | Total poultry population * | Poultry population produced by EthioChicken** |
| --- | --- | --- |
| Tigray | 6,190,640 | 2,384,858 |
| Amhara | 17,705,026 | 3,058,432 |
| Oromia | 19,014,114 | 5,355,333 |
| Southern Nations, Nationalities, and People’s region | 10,491,131 | 6,214,696 |
| Total of the four regions | 53,400,911 | 17,013,319 |
| Ethiopia | 56,056,778 | 17,013,319 |

*those data come from:

Central Statistical Agency of Federal Democratic Republic of Ethiopia. Agricultural sample survey 2017/18 [2013 E.C.], volume II, report on livestock and livestock characteristics. (2018) Available at: https://www.statsethiopia.gov.et/wp-content/uploads/2020/02/Agricultural-Sample-Survey-Livestock-Poultry-and-Beehives.pdf [Accessed November 2, 2021]

**Internal data from EthioChicken
