# Supplementary material for: Evaluation of Public–Private Partnership in the Veterinary Domain Using Impact Pathway Methodology: In-depth Case Study in the Poultry Sector in Ethiopia
Source: Front Vet Sci. 2022 Feb 22;9:735269. doi: 10.3389/fvets.2022.735269 (PMC8901995; doi:10.3389/fvets.2022.735269)
Supplement: Supplementary file 2 [file Table_2.DOCX]

**Supplementary Table 2.** **Number of participants of the participatory impact pathway evaluation are stakeholders from different administrative levels of the public-private partnership between EthioChicken and the Ethiopian government:** public and private partners of the partnerships, actors who influence it and actors impacted by it**.**

| Categories of actors | Actors | Administrative level | Semi-structured interviews  (individual and focus group) | Workshops | | |
| --- | --- | --- | --- | --- | --- | --- |
|  |  |  |  | 1st | 2nd | 3rd |
| *Private actors* | | | |  | | |
| Independent actors | Grower agents (growers of 45 days-old chickens) | District | 8 in one focus group | 3 | 3 | - |
|  | Village poultry development agents | Ward (kebelle) | 2 | 1 | 6 | - |
|  | Smallholders farmers (buyers of 45 days old chickens) | Ward (kebelle) | 19 and 4 in one focus group | 1 | 2 | - |
| EthioChicken | Managing director and sales manager | National | 2 | 2 | 2 | 12 |
|  | Farm hatchery, farm site, farm breeding and sale manager | Regional | 4 | 6 | 7 | - |
|  | Area sales manager, district (woreda) coodinator | District | 2 | 3 | 2 | - |
| Poultry Producers and Processers Association | Members of the association | National | 1 | - | 2 | 1 |
| Microfinance Institution | Staff of the Operation department | National and regional | 2 | 2 | 2 | 5 |
| *Public actors* | | | |  | | |
| Actors from public Veterinary Services and other actors of Ministry of Livestock and Fisheries* | Poultry production director, and Coordinator of public-private partnerships | National | 2 | 1 | 2 | 2 |
|  | Regional livestock officer | Regional | 1 | - | 4 | 4 |
|  | Head, vice head and livestock expert of districts | District | 3 | 3 | - | - |
|  | Development agents | Ward (kebelle) | 3 | 2 | 2 | - |
| Ministry of Health | Department of Public Health | National | - | - | 2 | 2 |
| Researchers | Social Scientist | International (ILRI) | 1 | - | - | - |
|  | Animal genetics and breeding |  | 2 | 1 | 1 | - |
|  | Veterinary Science, Animal production | National (Ethiopian University) | 1 | 1 | 1 | - |
| Pan-African Vaccines Control | Diagnostic department | International | 1 | - | 2 | - |
| Job Opportunity Creation Agency | Deputy director of agency | National | 1 | - | - | - |
|  | Officer in rural department | Regional | 1 | - | 2 | - |
| National Animal Health Diagnostic Investigation Center, public Veterinary Services | Associate researcher in virology, bacteriology and serology | National | 2 | - | 2 | 1 |
| National Veterinary Institute, public Veterinary Services | Head of research department | National | 1 | - | 2 | - |
| Veterinary Drug, Animal Feed, Administration Control Authority | Department of veterinary drug quality standard registration | National | 1 | - | 2 | 1 |
| Total | | | 52 and 12 in focus group | **26** | **48** | **18** |
|  |  |  | **64** |  |  |  |

*The Ministry of Livestock and Fisheries was merged with Ministry of Agriculture since April 2018.

**Supplementary Table 2 bis.** **Participants of the participatory impact pathway evaluation per region.** *SNNPr = Southern Nations, Nationalities, and People’s region*

| Categories of actors | Actors | Administrative level | Semi-structured interviews  (individual and focus group) | Region |
| --- | --- | --- | --- | --- |
| *Private actors* | | | |  |
| Independent actors | Grower agents (growers of 45 days-old chickens) | District | 8 in one focus group | Oromia |
|  | Village poultry development agents | Ward (kebelle) | 1 | Amhara |
|  |  |  | 1 | SNNPr |
|  | Smallholders farmers (buyers of 45 days old chickens) | Ward (kebelle) | 7 | Oromia |
|  |  |  | 3 | Tigray |
|  |  |  | 2 | Amhara |
|  |  |  | 7 + 4 in one focus group | SNNPr |
| EthioChicken | Farm hatchery, farm site, farm breeding and sale manager | Regional | 1 | SNNPr |
|  |  |  | 1 | Oromia |
|  |  |  | 1 | Tigray |
|  |  |  | 1 | Amhara |
|  | Area sales manager, district (woreda) coodinator | District | 1 | SNNPr |
|  |  |  | 1 | Tigray |
| Microfinance Institution | Staff of the Operation department | Regional | 1 | SNNPr |
| *Public actors* | | | |  |
| Actors from public Veterinary Services and other actors of Ministry of Livestock and Fisheries* | Regional livestock officer | Regional | 1 | SNNPr |
|  | Head, vice head and livestock expert of districts | District | 1 | Tigray |
|  |  |  | 1 | oromia |
|  |  |  | 1 | Amhara |
|  | Development agents | Ward (kebelle) | 2 | Oromia |
|  |  |  | 1 | Tigray |
| Job Opportunity Creation Agency | Officer in rural department | Regional | 1 | SNNPr |

*The Ministry of Livestock and Fisheries was merged with Ministry of Agriculture since April 2018.
