# Supplementary material for: Evaluation of Public–Private Partnership in the Veterinary Domain Using Impact Pathway Methodology: In-depth Case Study in the Poultry Sector in Ethiopia
Source: Front Vet Sci. 2022 Feb 22;9:735269. doi: 10.3389/fvets.2022.735269 (PMC8901995; doi:10.3389/fvets.2022.735269)
Supplement: Supplementary file 3 [file Table_3.DOCX]

**Supplementary Table 3. Summary of benefits and impacts generated by the public-private partnership and the related actors mentioned by the participants in this study.**

| Benefits | Impact | Actors impacted |
| --- | --- | --- |
| Improved individual and business confidence (sense of security; lower risks and stress; employment) | BUSINESS, TRUST | Private  -Farmers  -Grower agents  -Staff of EthioChicken  -Village poultry development agents |
| Improved reputation through quality product | BUSINESS, TRUST | Private  -EthioChicken  -Hendrix |
| Improved synergy between private and public sectors | TRUST | Private  -EthioChicken  Public  -National animal health diagnostic and investigation center  -Veterinary drug and animal feed and administration control authority  - Development agents (regional level) |
| Improved trust among the different actors of the supply chain (higher quality products) | TRUST | Private  -Farmers  -Grower agents |
| Improved competences (poultry production and health) | TRUST, HEALTH | Private  -Farmers  -Grower agents  -Staff of EthioChicken  -Village poultry development agents  Public  -Veterinary drug and animal feed and administration control authority  - Development agents (regional level) |
| Improved chicken production | ECONOMY, HEALTH | Private  -Farmers  -EthioChicken  -Grower agents  -Consumers  Public  -National government |
| Increased market access | ECONOMY, BUSINESS | Private  -EthioChicken  -Hendrix company  -National veterinary institute  -National crop producers |
| Improved poultry disease control | ECONOMY | Private  -Farmers  -Staff of EthioChicken  -Village Poultry Development Agent  Public  - National government  - Veterinary drug and animal feed and administration control authority  -Development agents |
| Improved profit/revenues | ECONOMY, SOCIETAL | Private  -EthioChicken  -National crop producers  -Farmers  -Grower agents  -Staff of EthioChicken  -Village poultry development agents  Public  -Government (national and regional)  -Microfinance institutions (public/private) |
| Increased employment | ECONOMY, SOCIETAL | Private  -Farmers  -Grower agents  -Staff of EthioChicken  -Village poultry development agents  Public  -Government (regional, national) |
| Improved livelihood | SOCIETAL | Private  -Farmers  -Staff of EthioChicken  -Village poultry development agent  Public  -Development agents (regional) |
| Empowerment of women | SOCIETAL | Private  -Farmers  -Staff of EthioChicken |
