# Supplementary material for: Evaluation of Public–Private Partnership in the Veterinary Domain Using Impact Pathway Methodology: In-depth Case Study in the Poultry Sector in Ethiopia
Source: Front Vet Sci. 2022 Feb 22;9:735269. doi: 10.3389/fvets.2022.735269 (PMC8901995; doi:10.3389/fvets.2022.735269)
Supplement: Supplementary file 4 [file Table_4.DOCX]

**Supplementary Table 4. Benefits, limits and solutions proposed during the second workshop for each actor involved in the national and regional public-private partnerships between the Ethiopian government and EthioChicken**.

*Sasso breed is a dual-purpose improved genetic breed from Hendrix genetics.

| **Actors** | **Benefits for the actors** | **Limits** | **Solutions (in regards with the partnership)** |
| --- | --- | --- | --- |
| **EthioChicken (private)** | **PPP National**  -Access to National Animal Health Diagnostic and Investigation Centre surveillance disease and training of the agents and farmers for free  -Loan to agents through Microfinance Institutions and Job opportunity creation agency | **PPP National**  -EthioChicken holds earned exclusivity of Sasso breed:   - stigmatization and suspicious feeling from competitors - non access to Poultry producer association=limit market access;   - No access to foreign exchange currency threatens the stability of the activity: | **PPP National**  -Exclusivity right of Sasso breed* is an asset to EthioChicken  -Access of improved genetics to other producers; Sasso has given improved genetics to farms in one region (which is directly under monitoring of Sasso genetic breeder).  -Poultry production needs to become a priority industry for Ministry of Finance and Economic Development to have access to foreign exchange currency /export market (**scenario 1**) |
|  | **PPP Regional**  -Increase market access (through development agent; Village poultry development agents);  -Increase market demand (satisfaction of the farmers);  -Increase reputation from quality products;  -Improved profits (chicken sales); | **PPP Regional**  - Instability of the market (fasting period) | -The government is promoting chicken meat consumption |
| **Government (national) (public)** | **PPP National**  -Increase in national chicken production (improved national economy) participates to the achievement of the Growth and transformation plan II.  -Increase in employment (young veterinarians enrolled by EthioChicken, agents and their paid staff, Village poultry development agents)  -Increase in profit through the sales of national vaccines (National veterinary institute)  -Increase sales of local crops to EthioChicken. | **PPP National**  -National economy protection (limited foreign exchange currency import) that threat the stability of the actions and therefore its long-term impacts | **PPP National**  -Poultry production needs to become a priority industry for the government to have access to foreign exchange currency/export market; |
| **Government (regional) (public)** | **PPP Regional**  -Increase in national chicken production (improved regional economy)  -Improved trust from poultry consumers (quality chickens + prices regulation)  -Increased in regional budget (from share of business profit with EthioChicken due to the rent of regional farm )  -Improve local employment (Village poultry development agents, grower agents) | **PPP Regional**  -Village poultry development agents threaten the PPP at regional level with livestock offices and development agents (stability of this specific partnership) | **PPP Regional**  -EthioChicken to function more independently at local level could be positive (no influence on the overall impact); this part of the partnership could be seen as transitional (which is already the case in some regions) |
| **Government (development agents) (public)** | **PPP Regional**  -Increase income;  -Improve trust by farmers (higher competences) | **PPP Regional**  -The knowledge of development agents about poultry management is low  -Sort of negative relationship between development agents and agent | **PPP National and regional**  -Training of the development agent |
| **Grower agents (45 day old chicken producers) (private)** | **PPP Regional**  -Improved chicken production (better production; lower mortality);  -Improved employment access (lower investment risks);  -Improved security feeling (lower risks and stress);  -Increased incomes (better production; market access guarantee);  -Improved competences (poultry production and health) trough training | **PPP National**  -Limited access to loan and capital  **PPP Regional**  - Market unstability (consumption problem due to cultural fasting practices)  -High price of inputs (feed)  -Delay in money collection by development agent and Village poultry development agent | **PPP National**  -Access to more capital  -The government is promoting the consumption of chicken meat |
| **Farmers (private)** | **PPP Regional**  -Increase in chicken production (better production; lower mortality);  -Increased incomes;  -Improve security feeling (lower risks and stress);  -Improve trust by consumer (quality products);  -Improved competences (poultry production and health) | **PPP National**  -Limited access to feed  -Limited access to health service  -Limited access to land  **PPP Regional**  -Low management capacity  -Low breeding identification (some farmers prefer meat, some eggs)  -Market unstability (consumption problem due to cultural fasting practices) | **PPP National**  -Establishment of feed manufacturing enterprises by private sector with support from government  -Increase poultry feed availability (feed shops in localities) by privates with support from government.  -Access to more capital  -Improve development agent competencies who can help farmer  -The government is promoting consumption of chicken meat |
| **National crop producers (private)** | **PPP National**  -Increased market demand and increased income | **PPP National**  -Sustainability of the activity is threatened due to food supply shortage (not enough crops compared to the demand) and problem to land access | **PPP National**  -The government could provide incentives for the sector to grow to increase maize and soya bean production  - The government could use prospective crop production estimates to meet the local industry consumption so that only excess product is exported |
| **National Vaccine Institute (public)** | **PPP National**  -Increased incomes for National veterinary institute (huge demand of vaccines from EthioChicken) | **PPP National**  - High cold chain constraints and reagents supply issues (no foreign exchange currency access) for National veterinary institute  -Local vaccines are expensive (3 to 4x more than international vaccines)  -Conflict of interest among different private vaccines producers | **PPP National**  -Poultry production needs to become a priority industry for Ministry of Finance and Economic Development to have access to foreign exchange currency/export market;  -National veterinary institute could only produce vaccines that are not global vaccines internationally supplied |
| **Microfinance institutions (public-private) and Job opportunity creation agency (public)** | **PPP National**  -Strengthening of their activity and results/impact: higher income.  -better results (lower risk business; training supported by EthioChicken) | **PPP National**  -Their knowledge about poultry management is low and the credits give to farmers is low  -Sometimes the young agent do not have the capacity to reimburse the loan | **PPP National**  -Increase knowledge of Microfinance Institutions in poultry management and increase the loan amount |
| **Hendrix genetics (private)** | **PPP National**  -Improved reputation (from performance of their breed; linked to quality production)  **PPP Regional**  -Improved trust of their breed by farmers: could lead to other market access (e.g. Kroiler, Hubbard, Tetra, Aviagen Range Red) | **PPP National**  -Problem of importation because of Avian Influenza in France so EthioChicken have to import from Brazil  -Exclusivity contract with EthioChicken = limited Ethiopian market | **PPP National**  -Access of improved genetics to other producers but risk of losing market; |
| **National Animal Health Diagnostic and Investigation center (public)** | **PPP National**  -Facilitate National animal health diagnostic and investigation centre agent surveillance and control activity | **PPP National**  - Diagnostic kit test supply issues for National Animal Health Diagnostic and Investigation Centre no foreign exchange currency access | **PPP National**  -Poultry production needs to become a priority industry for Ministry of Finance and Economic Development to have access to foreign exchange currency/export market; |
| **Other poultry producers (private)** | **PPP National**  -Possible improvement of the sale of their chicken (growth poultry industry market + consumer demands) | **PPP National**  Loss of production market (higher risk business for agents and farmers), agents goes to EthioChicken because of better breed (faster and more resistant to disease and technical support from Vet) | **PPP National**  -Access of improved genetics to other producers and adoption of EthioChicken model |
| **Poultry producer association (private)** | **PPP National**  -Increased power due to stronger poultry industry (through government action and EthioChicken business) | **PPP National**  -EthioChicken is not in the association because other poultry producers have suspicious feeling about EthioChicken and its exclusivity right on Sasso breed;   - Weakness their power (lower lobbying options) | **PPP National**  -Both parties agree (public and EthioChicken) that it would strengthen the poultry industry if EthioChicken was part of the poultry producer association - EthioChicken can introduce other producers to other breeding houses with similar genetics but the breeding houses themselves set criteria for supply to a breeder farm., |
