# Supplementary material for: Evaluation of Public–Private Partnership in the Veterinary Domain Using Impact Pathway Methodology: In-depth Case Study in the Poultry Sector in Ethiopia
Source: Front Vet Sci. 2022 Feb 22;9:735269. doi: 10.3389/fvets.2022.735269 (PMC8901995; doi:10.3389/fvets.2022.735269)
Supplement: Supplementary file 5 [file Data_Sheet_1.DOCX]

**Supplementary file 1. Checklists used for the individual semi-structured interviews of the stakeholders of the case study.**

1. Check list for actors at the conception of the PPP

| **THEMES** | **TOPICS** | **QUESTIONS** |
| --- | --- | --- |
| BUILDING of the PPP | Recruitment of partners | 1-How did you define that a partner is good to work with?  2-What are you expectations from each partner? |
|  | Motivations to participate |  |
|  | Commitment of partners |  |
|  | Organization of the public-private partnership | Is there a formal document about all your partnerships process? |
|  | Roles and responsibilities |  |
|  | Time commitment |  |
|  | legality of the partnership |  |
|  | Risk identification | 1-Did you have some apprehensions before weaving this partnership? Why?  2-What attitude do you have in front of these kinds of apprehensions? |
|  | Risk awareness |  |
|  | Risk allocation |  |
|  | Performance indicators | How will you know that this partnership run well? Why? |
| FUNCTIONING of the PPP | Collaboration | 1-Tell me about the functioning of this PPP?  2-how do you make it work?  What is your perception of this functioning?  3-Is there something that you could suggest to make it more efficient? |
|  | Communication |  |
|  | Management /Leadership |  |
|  | Governance structure |  |
|  | Transparency |  |
|  | Actors involvement |  |
|  | Promptness |  |
|  | Trust and respect |  |
|  | Risk management |  |
| OUTPUTS of the PPP | Action plans and interventions | Could you please tell me what these partnerships brought (output) to your Enterprise? |
|  | Partnership's goals | Did these contributions from these partnerships meet your expectation? Why? |
|  | Impact (what, where, how, whom and when) | What are the benefits, chages or impacts bought by this PPP? |
|  | Perceived efficiency (resource efficiency to meet objectives) |  |
|  | Benefit and sustainability |  |
|  | Policy changes |  |
|  | Changes in the physical environment |  |
|  | Changes in the social environment |  |
|  | Changes in health indicators |  |
|  | Changes in financial income |  |
|  | Others changes |  |

1. Check list for the operational public and private partners of the PPP

| Themes | Questions |
| --- | --- |
| Poultry production | 1. Could you tell me about the importance of poultry production? 2. Could you tell me about any issues in poultry farming?   *-Could you tell me about the situation before?*  *-Why is it better/ worse now?* |
| Role in the PPP and in EthioChicken mode, | 1. What is your involvement in the PPP between EthioChicken and the Ethiopian government ?  - How does the PPP works? EthioChicken model? What do they do exactly? - What is your role? Who are you in contact with? - Why did you accept working with them? - Do you have any agreement with the different people you work with in the program? With the poultry producers? Which kind of agreement? (check for any written agreement) |
| Interactions with other stakeholders | 1. Could you tell me about your relationship with the farmers, the government, the development agents and the village poultry development agent?  - Who do you work with the most? Why? How? |
| Benefits of the PPP | 1. What do you get (as benefit) from this model of EthioChicken and Ethiopian government? Comparing to the past? 2. What does this program brings to your community? The poultry producers? Others? (e.g. women groups?) |
| Limits and scenario of improvement | 1. Is there any issue? Which services do you want EthioChicken or Ethiopian government to improve?  - Why? - How?  1. If you had a message to address to EthioChicken Company, what will you say to them? Why? |

1. Checklist for the actors who adopted the model (farmers)

| Themes | Questions |
| --- | --- |
| Poultry production | 1. Could you describe your poultry production activity?   -Who is taking care of your production? Could you tell me about any issues you have with your poultry farming? What difficulties do you encounter?  2) Could you tell me about the importance of poultry production for you? What do you get from breeding chickens? Why is it important for you? what do you do with this money? (e.g. get children to school; buy school furniture’s; buy things for the house…) |
| Participation in the EthioChicken model | 3) Could you tell me about your involvement with EthioChicken?  - How does it work for you (Ethiockicken program)? Who are you in contact with (who sell them the chickens and help them with their production)?  - What do they do exactly?  - Why did you accept working with them?  - Do you have any written agreements? Which ones? Other type? |
| Interactions with other stakeholders | 4) Are you involved in producer association? Which ones?  5) Could you tell me about your relationship with the agents/ the development agents and the village poultry development agent?  - Who do you work with the most? Why? |

| Benefits of the PPP | 6) What do you get (as benefit) from this program of EthioChicken? Comparing to the past (or before)? |
| --- | --- |
| Limits and scenario of improvement | 7)Is there any issues? What do you want EthioChicken to improve as service?  - Why?  - How?  8) If you had a message to address to EthioChicken Company, what will you say to them? Why? |
