# Supplementary material for: Evaluation of Public–Private Partnership in the Veterinary Domain Using Impact Pathway Methodology: In-depth Case Study in the Poultry Sector in Ethiopia
Source: Front Vet Sci. 2022 Feb 22;9:735269. doi: 10.3389/fvets.2022.735269 (PMC8901995; doi:10.3389/fvets.2022.735269)
Supplement: Supplementary file 6 [file Data_Sheet_2.DOCX]

**Supplementary material 2. The different codes that emerged from the reading of the transcripts and used for the data analysis.**

PPP: public-private partnership

| **Codes** | **Sub codes** |
| --- | --- |
| Context | Poultry production in Ethiopia |
|  | Story of the public-private partnership |
| Partners of the PPP | Ethiopian Government |
|  | Business partner |
|  | Independent private actors |
| Functioning of the PPP | PPP process and contracts |
| Importation of inputs | Importation and input pathway: Chicken |
|  | Importation and input pathway: Vaccines |
|  | Importation and input pathway: feed |
| Trainings organized in the PPP | Training pathway: public veterinarians |
|  | Training pathway: private veterinarians |
|  | Training pathway: farmers |
| Production and delivery of the day old chick and 42 days old chicks | Production Pathway (AGENT) |
|  | Delivery pathway to grower agents and intermediary |
|  | Delivery at farmers' level |
| Benefits of the PPP | Women empowerment/livelihood |
|  | Employment |
|  | Competencies; improvement of services |
|  | Food security; livestock productivity; disease control |
|  | Profit/revenue; optimisation/efficiency |
|  | Collaboration / trust between government and private |
| Limits and solutions proposed | Problem of importation |
|  | Solution proposed / recommendation: importation |
|  | Low considering of poultry sector by the government |
|  | Solution proposed / recommendation: government |
|  | Problem of capital (farmers; agent) |
|  | Solution proposed / recommendation: capital |
|  | Problems of poultry consumption/ market |
|  | Solution proposed / recommendation: consumption market |
|  | Problems of poultry production |
|  | Solution proposed / recommendation: production |
|  | Problem of transportation ; input |
|  | Solution proposed / recommendation: input |
|  | Problem between actors |
|  | Solution proposed / recommendation: problem between actors |
|  | Solution proposed / recommendation: other |
| Added value of the PPP | Private and Public Point of view |
|  | Public Point of view |
|  | Private Point of view |
|  | Farmers Point of view |
| Key success factors of the PPP | Conditions for success/ key success factors |
