# Supplementary material for: Evaluation of Public–Private Partnership in the Veterinary Domain Using Impact Pathway Methodology: In-depth Case Study in the Poultry Sector in Ethiopia
Source: Front Vet Sci. 2022 Feb 22;9:735269. doi: 10.3389/fvets.2022.735269 (PMC8901995; doi:10.3389/fvets.2022.735269)
Supplement: Supplementary file 7 [file Data_Sheet_3.DOCX]

**Supplementary file 3. The definitions of inputs, outputs, outcomes, first level impacts and second level impacts.**

PPP: public-private partnerships

| Economic impacts of PPPs | Economic impact refers to stronger national economy through the positive impact of improved animal health and other activities in the veterinary domain on livestock productivity, rural livelihoods, and domestic and export competitiveness of the sector [1]. |
| --- | --- |
| Buisness impacts of PPPs | Business impact is related to reduced business risk and increased opportunities (or the enabling environment) for innovative approaches and sustainable solutions for the private sector: positive working relationships with the public sector at national and local levels; inﬂuence and insight from the Government policy process, leading to policies more supportive of the private sector and the ability to align business strategies to work with Government priorities; access for private partners to Government contracts and additional income [1]. |
| Societal impact | PPPs also contributes to addressing societal issues: livestock is a major asset, important to the livelihood of economically vulnerable communities and often under responsibility of women and youngsters; additional revenues provided by improved animal health will directly contribute to reducing gender inequality and improving the lives and access to education of the poorest [1]. |
| Impact on trust | Impact on trust PPPs in the veterinary domain means a greater trust between the Government and private partners: high quality services and other activities delivered through PPP improve the reputation and trust of the service providers (both public and/or private) by end-users. Stakeholders, including end users of improved services, are supportive of the Government’s policies and approach, including the use of PPP. Joint development of animal health and welfare policies, such as disease control programs or enabling exports, lead to wider stakeholder support [1]. |
| Impact on health | PPP can improve veterinary services which will reduce zoonotic disease risks and increase food security by providing access to affordable proteins for the poorest communities leading to health impacts [1]. |

1. World Organisation for Animal Health. The OIE PPP Handbook: guidelines for public-private partnerships in the veterinary domain. 2019 [cited 11 Mar 2020]. Available: https://www.oie.int/publicprivatepartnerships/ppp/en/Handbook_en.html
